# Codebook-guided multi-cue normality modeling for pulmonary CT anomaly detection

**DOI:** 10.3389/fmed.2026.1927996

**Published:** 2026-09-25

**Authors:** Huizhong Wang

**Affiliations:** School of Mathematics and Statistics, Faculty of Science, The University of Sydney, Sydney, NSW, Australia

**Keywords:** anomaly detection, lesion localization, patient-level evaluation, pulmonary CT, quantization distance, transformer, vector quantization

## Abstract

**Introduction:**

Automated anomaly detection from chest computed tomography (CT) may support research on pulmonary image triage without requiring exhaustive abnormal annotations. Supervised methods depend on costly expert labels, while many existing normal-only methods rely predominantly on a single anomaly cue. In pulmonary CT, heterogeneous lesion appearance and variation across slices and acquisition sources may reduce the reliability of single-cue scoring. This study develops a codebook-guided framework that integrates complementary cues for abnormality detection and spatial heatmap generation.

**Methods:**

The framework combines residual vector-quantized reconstruction, transformer-based token discrimination learned through codebook-guided latent perturbation, and quantization-distance scoring. The primary benchmark combined three public CT datasets. Slice-level evaluation used a visually screened, lesion-enriched subset, whereas patient-level evaluation retained all valid lung-containing slices and aggregated the highest 10% of slice scores by their mean. Patient-level thresholds were calibrated using normal validation patients only. Quantitative localization was additionally evaluated on 100 LIDC-IDRI cases containing nodules marked by at least three radiologists.

**Results:**

The proposed method achieved a slice-level AUROC of 0.9218. At the patient level, it achieved an AUROC of 0.9187±0.0123, balanced accuracy of 0.7521±0.0354, and F1-score of 0.9297±0.0052; the patient-stratified bootstrap 95% confidence interval for AUROC was 0.8786–0.9727. Disease-wise patient-level AUROC ranged from 0.8470 for COVID-19 to 0.9747 for community-acquired pneumonia. On the external LIDC-IDRI localization cohort, the proposed method achieved a P-AUROC of 0.9155, the highest observed value among the evaluated methods.

**Discussion:**

The findings indicate that multi-cue normality modeling remains effective after patient-level aggregation and provides quantitative localization capability on an external nodule cohort. Same-source comparisons within COVID-CT-MD reduce, but do not eliminate, concern about dataset-source confounding. Because the study used retrospective public datasets and lacked prospective patient-level external validation, the results should be interpreted as methodological evidence rather than clinical validation.

## 1 Introduction

Pulmonary diseases, including pneumonia, lung cancer, tuberculosis, and other infectious or inflammatory lung conditions, remain a major clinical burden and often require timely imaging-based assessment. Computed tomography (CT) is widely used for pulmonary disease screening, diagnosis, and follow-up because it provides high-resolution cross-sectional views of lung anatomy and can reveal subtle pathological patterns that may be difficult to identify using planar imaging. In recent years, deep learning has achieved substantial progress in medical image analysis (1), including pulmonary disease classification, COVID-19 screening, pneumonia assessment, and lesion segmentation from chest CT images (2–6). These methods have shown the potential to assist radiologists by improving screening efficiency and highlighting suspicious regions for clinical review.

However, pulmonary CT interpretation remains challenging in practice. Abnormal findings may appear as large diffuse opacities, small focal nodules, consolidations, infection-related textures, or subtle structural changes. Their appearance can also vary across scanners, acquisition protocols, disease stages, and patient populations. In addition, a single CT volume may contain many axial slices, only some of which show abnormal patterns. This motivates automated screening models that can rank CT slices by abnormality and provide spatial heatmap cues for further expert review, rather than directly producing a final clinical diagnosis.

Most existing pulmonary CT analysis models are developed under supervised learning settings. These models usually require labelled abnormal cases, and stronger forms of supervision may require lesion bounding boxes, pixel-level masks, or other expert annotations. Preparing such datasets is time-consuming and expensive, and the annotation process depends heavily on specialist knowledge (7). In addition, supervised models are usually optimized for a predefined set of disease categories. This closed-set formulation may limit their ability to respond to rare, atypical, or previously unseen abnormalities. The imbalance between normal and abnormal cases can further increase the difficulty of supervised model development (8).

Normal-only anomaly detection provides a promising alternative. Instead of learning a fixed set of disease categories, the model learns the distribution of normal data and treats strong deviations from this distribution as potential anomalies. This setting is well aligned with medical screening scenarios, where normal data are usually easier to define and collect, while abnormal patterns are heterogeneous and difficult to exhaustively enumerate. In pulmonary CT, such a model is not intended to replace clinical diagnosis. Rather, after appropriate external and prospective validation, it could support research-oriented triage by flagging suspicious images and providing spatial heatmaps for expert review.

Although unsupervised anomaly detection has been studied in several medical imaging domains (9–13), pulmonary CT anomaly detection has several specific challenges. First, normal lung appearance varies significantly across axial slices because lung size, anatomical structure, and tissue texture change along the superior-inferior direction. Second, pulmonary abnormalities are highly heterogeneous: some are compact and localized, while others are diffuse, scattered, or low contrast. Third, some lesions are visually similar to normal anatomical structures, such as vessels, bronchi, or local texture variations, making spatial localization difficult without pixel-level supervision.

These characteristics make single-cue anomaly detection unreliable. Existing normal-only anomaly detection methods often depend on one dominant anomaly signal, such as image-level reconstruction error, pretrained feature distance, density estimation, or synthetic anomaly classification. However, in pulmonary CT, each of these signals may fail under different conditions. Reconstruction residuals can provide fine spatial detail, but they may also be noisy and sensitive to normal anatomical variation. Feature-distance methods can provide stronger embedding-level comparison, but they may suffer from domain mismatch when natural-image pretrained backbones are transferred to grayscale CT images. Synthetic anomaly learning can introduce useful self-supervision, but image-space pseudo anomalies may not match realistic pulmonary lesion morphology.

Therefore, robust pulmonary CT anomaly detection should not rely on a single anomaly cue. Instead, normality should be modeled through complementary evidence from reconstruction discrepancy, contextual token consistency, and latent codebook deviation. This study addresses this gap by developing a codebook-guided multi-cue normality modeling framework, where a shared normal codebook provides the common basis for reconstruction, latent token perturbation, and quantization-distance-based anomaly scoring.

Motivated by the above observations, this study develops a normal-only pulmonary CT anomaly detection framework. The model is trained using only normal lung CT slices and is evaluated on multiple pulmonary abnormality categories. The key design is to use a shared residual vector-quantized normal codebook as the central representation. This codebook supports three complementary functions. First, it constrains the latent representation for reconstruction and helps model normal lung appearance through discrete code vectors (14). Second, it provides candidate codebook features for latent token perturbation, allowing a transformer-based branch to learn token-level contextual inconsistency. Third, it enables quantization-distance scoring by measuring the discrepancy between encoder features and their quantized counterparts. During inference, these cues are fused to produce a slice-level anomaly score and a spatial heatmap.

The main contributions of this study are summarized as follows:

- From the application perspective, this study constructs and evaluates a chest CT evaluation cohort covering five pulmonary abnormality categories, including COVID-19, community-acquired pneumonia (CAP), adenocarcinoma, nontuberculous mycobacterial pulmonary disease (NTM), and tuberculosis (TB). Under a normal-only slice-level screening setting, the proposed method is compared with representative anomaly detection baselines and achieves stronger detection performance. The generated spatial heatmaps also provide localization cues that highlight suspicious lung regions for qualitative review.
- From the algorithmic perspective, this study develops a codebook-guided multi-cue normality modeling framework that integrates residual vector-quantized reconstruction, codebook-based transformer token discrimination, and quantization-distance scoring. Built around a shared normal codebook, these components capture complementary information from pixel-level reconstruction discrepancy, token-level contextual inconsistency, and latent codebook deviation.

## 2 Related work

### 2.1 Supervised pulmonary CT analysis

Deep learning has been widely applied to pulmonary CT analysis. Existing studies have explored CT-based pneumonia screening, COVID-19 classification, multi-slice disease assessment, and lesion segmentation (2–6). Classification-based methods aim to predict disease labels from CT slices or volumes, while segmentation-based methods delineate infection regions, nodules, or other lesion areas when pixel-level annotations are available. These supervised methods can achieve strong performance when sufficient labels are available and when the target disease categories are clearly defined.

However, supervised pulmonary CT analysis depends strongly on annotated datasets. Image-level labels are less expensive than lesion annotations, but they still require diagnostic confirmation. Bounding boxes and pixel-level masks are more informative for localization, but they are substantially more costly to obtain and require specialist expertise. In addition, supervised models are usually trained for a fixed list of categories. This makes them suitable for known disease recognition, but less suitable for open-ended screening scenarios where rare, atypical, or unseen abnormalities may appear. These limitations motivate normal-only anomaly detection, where abnormal examples and lesion-level annotations are not required during training.

### 2.2 Medical anomaly detection under normal-only training

Unsupervised or normal-only anomaly detection aims to model normal data and identify samples that deviate from normality. This assumption has motivated a range of medical anomaly detection methods based on GANs, autoencoders, variational models, and other generative frameworks. Early studies applied these ideas to retinal imaging, brain MRI, and chest radiographs (9–12). More recent benchmark efforts, such as BMAD, further emphasize the need for standardized datasets, evaluation protocols, and fair comparison across medical anomaly detection methods (13).

For pulmonary imaging, normal-only anomaly detection is particularly relevant because the goal of screening is often to flag suspicious cases rather than to assign every image to a precise disease category. Nevertheless, pulmonary CT differs from many natural-image anomaly detection settings and from other medical imaging modalities. Normal anatomy changes across axial slices, and abnormal patterns can be subtle, diffuse, or visually close to surrounding tissue. As a result, both image-level detection and spatial heatmap generation remain challenging.

### 2.3 Reconstruction-based normality modeling

Reconstruction-based methods are among the most common approaches for medical anomaly detection. They train an autoencoder, variational autoencoder, GAN-based model, or diffusion model on normal data and use reconstruction error as the anomaly signal (9–11, 15–17). The key assumption is that a model trained only on normal samples should reconstruct normal structures well but fail to reconstruct abnormal regions.

This assumption is useful but not always reliable. In practice, abnormal regions may still be reconstructed if the decoder has strong generalization ability or if the lesion shares similar low-level structures with normal tissue. As a result, reconstruction residual maps may contain responses from normal anatomical variation while missing subtle abnormal regions. This issue is particularly important in pulmonary CT, where vessels, bronchi, and normal texture variations can resemble lesion-like patterns, and where many abnormalities are low contrast.

Vector-quantized representations provide a way to constrain the latent space through a discrete codebook (14). Instead of relying only on continuous latent features, a VQ-based model maps encoder outputs to learned code vectors. This can help form a structured representation of normal appearance. In this study, residual vector quantization is used to build a CT-adapted normal codebook. The motivation is not only to improve reconstruction, but also to provide a shared latent basis for token perturbation and quantization-distance scoring.

### 2.4 Feature-based and distribution-based anomaly detection

Another family of anomaly detection methods operates in feature space rather than directly in image space. These methods usually extract local embeddings from normal training images and then compare test features with normal feature statistics or stored normal prototypes. Representative approaches include PaDiM and PatchCore (18, 19). PaDiM models patch-level feature distributions using multivariate Gaussian statistics, while PatchCore constructs a memory bank of representative normal patch features and detects anomalies through nearest-neighbor distance (19). These methods can provide strong local comparison and are effective in many anomaly detection settings. However, their performance depends heavily on the quality and domain relevance of the extracted features.

Knowledge distillation methods provide another feature-level strategy. They train a student network to reproduce the representations of a teacher network on normal data, and use the discrepancy between teacher and student responses as the anomaly signal (20). Such methods can detect abnormal regions because the student network is expected to mimic the teacher well on normal samples but may produce larger feature discrepancies on abnormal samples. Nevertheless, many feature-based approaches rely on backbones pretrained on natural images. When transferred to grayscale CT images, these features may not fully capture domain-specific pulmonary texture and anatomical structure, which can limit localization precision for subtle or low-contrast lesions.

Distribution-based methods explicitly estimate the normal feature distribution. Normalizing-flow-based methods, such as CS-Flow, learn an invertible mapping of normal multi-scale features and identify low-likelihood regions as anomalies (21). Compared with memory-bank or distillation methods, normalizing flows provide an explicit density estimation mechanism. However, in pulmonary CT images, the normal feature distribution can vary substantially across slices and anatomical regions, while lesions can be diffuse or visually similar to normal tissue. Therefore, distribution-based responses may still be unstable when the abnormality does not form a clearly separable low-likelihood pattern.

### 2.5 Synthetic anomaly learning and token-level discrimination

Synthetic anomaly learning provides a way to train anomaly detectors without real lesion annotations. The basic idea is to create artificial abnormal patterns from normal samples and train a model to distinguish normal data from the synthesized abnormal data. CutPaste generates pseudo anomalies by cutting and pasting image patches, and then learns representations for anomaly detection through this self-supervised discrimination task (22). This strategy is simple and effective in some visual anomaly detection settings, but image-space patch manipulation may introduce artificial boundaries or texture discontinuities that do not match realistic pulmonary lesion morphology.

More recent work has explored synthetic anomaly learning in feature space. SimpleNet extracts pretrained local features, injects Gaussian noise into the feature embeddings to synthesize anomalous features, and trains a lightweight discriminator to separate normal features from synthetic abnormal ones (23). Although SimpleNet is often discussed together with feature-based anomaly detection because it uses pretrained representations, its training signal is closely related to feature-space synthetic anomaly learning. In medical image anomaly detection, SyNet further explores the idea of noise-based anomaly synthesis through a noise synthesis network, showing that synthetic feature or noise perturbation can be adapted to medical imaging scenarios (24).

These methods suggest that synthetic anomaly learning can provide useful supervision when real abnormal annotations are unavailable. However, the design of the synthetic anomaly is critical. If the perturbation is too different from real lesions, the model may learn artificial shortcuts rather than clinically meaningful abnormality cues. This issue is especially important for chest CT, where lesions can be subtle, low contrast, or visually close to normal anatomical structures.

The transformer branch in this study follows the general motivation of feature-space synthetic anomaly learning, but implements it through a codebook-guided token discrimination task. Instead of adding visible artifacts in image space, selected latent tokens are replaced by alternative candidates from the learned normal codebook. The replacement token may still be a normal code vector by itself, but it can become contextually inconsistent after being inserted into a different latent location. In this way, anomaly synthesis is formulated as a token-level contextual discrimination problem, encouraging the transformer to learn whether each latent token is consistent with the surrounding normal structure.

### 2.6 Positioning of this study

The reviewed literature suggests that no single anomaly cue is sufficient for robust pulmonary CT anomaly detection. Reconstruction-based methods provide intuitive residual maps, but they may suffer from overgeneralized reconstruction and noisy responses to normal anatomical variation. Feature-based methods provide embedding-level comparison, but they may suffer from domain mismatch when natural-image pretrained networks are transferred to CT images. Distribution-based methods model normal feature likelihood, but their responses may be unstable across heterogeneous pulmonary patterns. Synthetic anomaly learning provides self-supervision without lesion annotations, but image-space synthetic artifacts may not align with real pulmonary lesions.

This study is positioned between these directions. Instead of relying on reconstruction error, pretrained feature distance, or image-space pseudo anomalies alone, it uses a shared residual vector-quantized codebook as the central representation for normality modeling. The codebook supports three complementary anomaly cues: reconstruction discrepancy, transformer-based contextual token inconsistency, and quantization-distance-based latent deviation. The final anomaly score fuses these cues to produce detection results, while the spatial heatmap provides qualitative localization cues for suspicious regions. This positioning motivates the methodology developed in the following section.

## 3 Materials and methods

### 3.1 Overview

The proposed framework is designed for lung CT anomaly detection under a normal-only training setting. Given a preprocessed lung-region CT slice $x\in R^{1\times H\times W}$, where H=W=128, the goal is not to classify a fixed set of known diseases, but to learn the normal pulmonary appearance and detect test images that deviate from this normal pattern.

As shown in Fig. 1, the framework contains three complementary branches. The first is a residual vector-quantized reconstruction branch, which learns a CT-adapted normal latent codebook and reconstructs lung CT images from quantized normal features. This branch provides a pixel-level reconstruction residual cue. The second is a quantization-distance branch, which measures how far each continuous latent token deviates from its quantized representation in the learned normal codebook space. This branch provides a token-level manifold deviation cue. The third is a transformer-based token discrimination branch, which is trained with codebook-based latent anomaly synthesis. Instead of generating artificial anomalies in image space, this branch perturbs selected latent tokens in the quantized feature space and learns whether each token remains consistent with its surrounding normal context.

**Figure 1:**
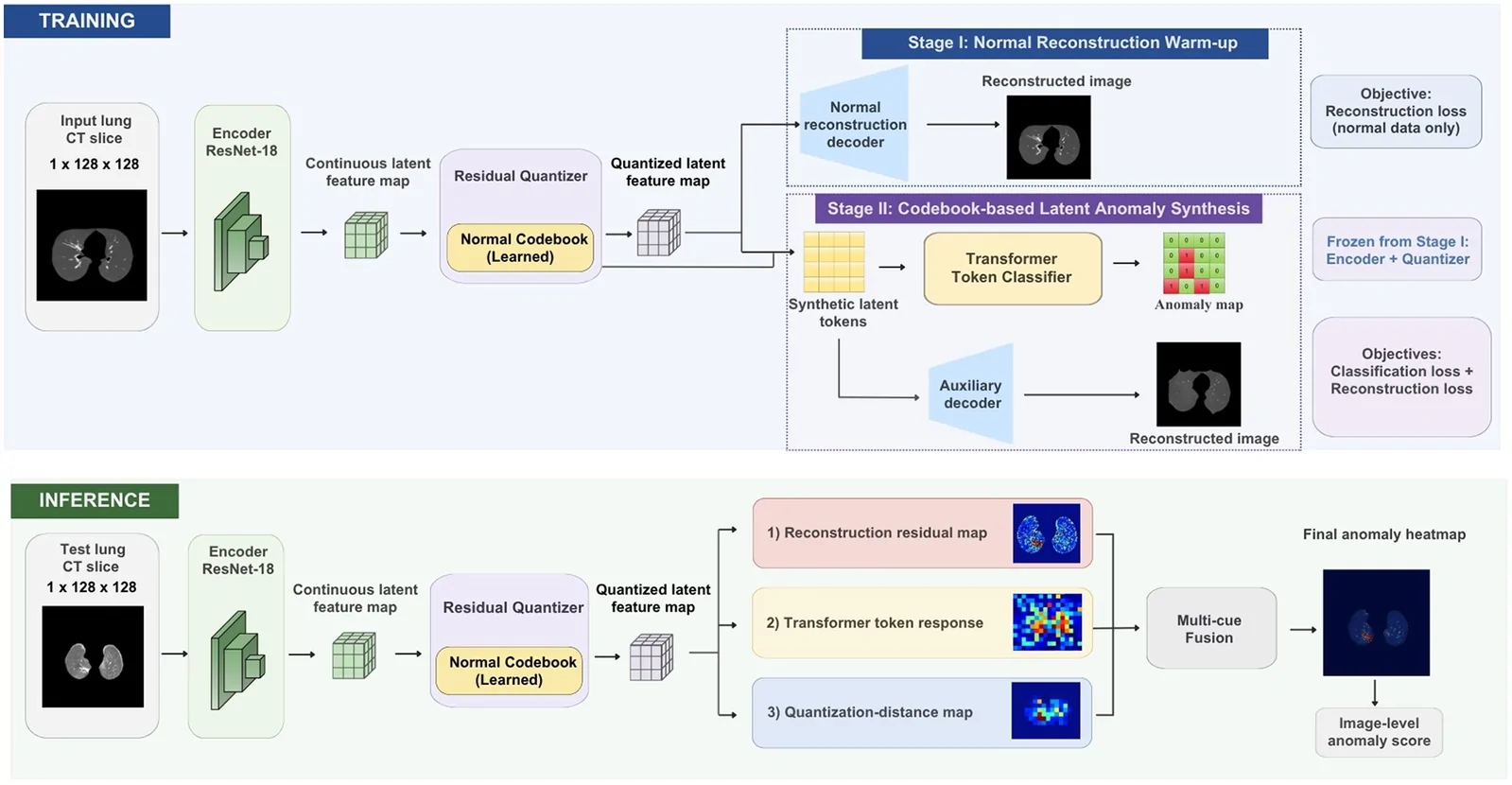
Overall framework of the proposed lung CT anomaly detection method. The model first learns a normal latent codebook from normal CT slices, and then combines reconstruction residuals, transformer token responses, and quantization-distance cues for anomaly scoring during inference.

The motivation for this design is that a single anomaly cue is often insufficient for lung CT anomaly detection. Reconstruction residuals can provide fine spatial details, but may be noisy or affected by normal anatomical variations. Transformer token responses can capture higher-level contextual inconsistency, but may be spatially coarse. Quantization-distance responses measure deviation from the learned normal codebook manifold and can strengthen suspicious latent regions. During inference, these three cues are fused to produce the final anomaly heatmap and slice-level anomaly score.

### 3.2 Residual vector-quantized reconstruction branch

As illustrated in Fig. 2, the reconstruction branch provides the foundation of the framework. Its purpose is to learn a discrete latent representation of normal lung CT appearance through residual vector quantization. Unlike using a fixed pretrained natural-image feature extractor, the encoder is further optimized on normal lung CT reconstruction, allowing the latent representation to become better adapted to the CT domain.

**Figure 2:**
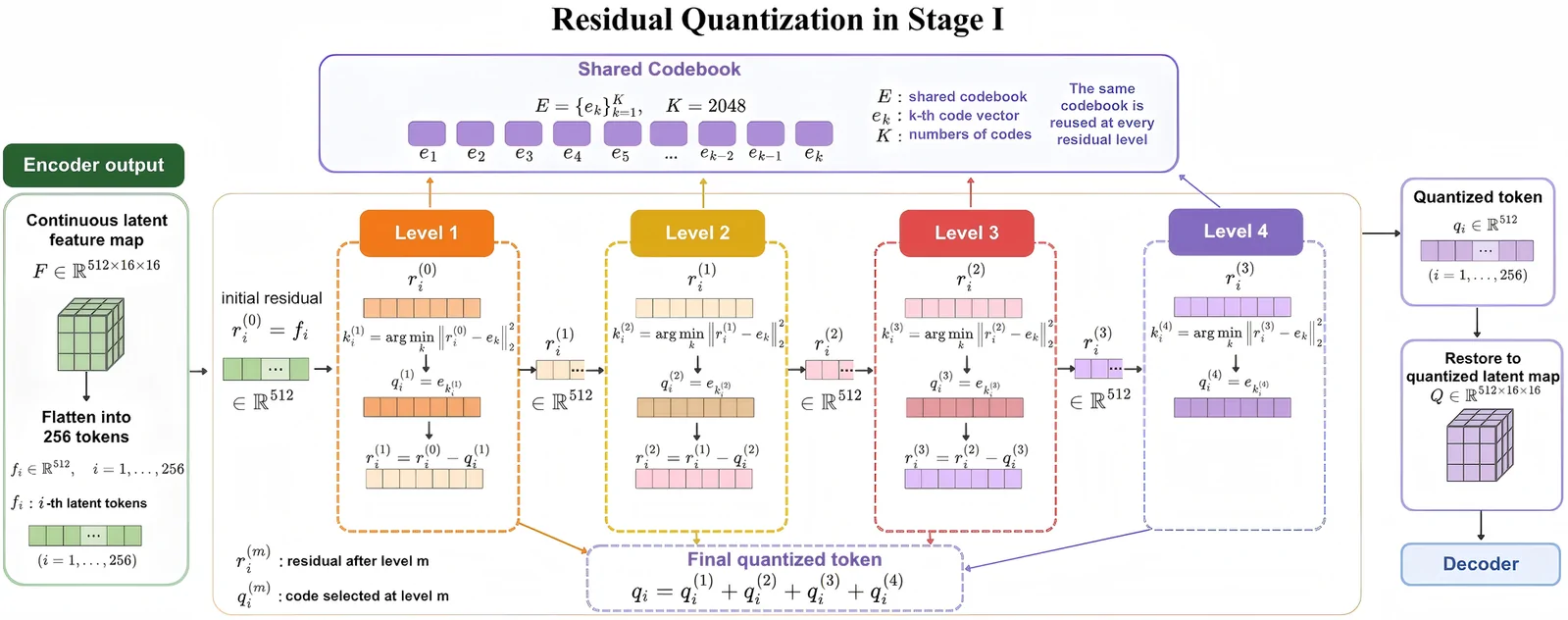
Residual quantization mechanism used in the reconstruction branch. The continuous encoder feature map is flattened into latent tokens. Each token is progressively approximated through multiple residual quantization levels using a shared normal codebook.

Let $E_{\theta }$ denote the encoder. Given an input image x, the encoder produces a continuous latent feature map

$$F=E_{\theta }(x),\;F\in R^{512\times 16\times 16}.$$ (1)

This feature map is flattened into N=256 latent tokens:

$$F=\{f_{i}{\}}_{i=1}^{N},\;f_{i}\in R^{512},\;N=16\times 16.$$ (2)

Each token represents a local latent region of the input CT slice.

To discretize these latent features, residual quantization is applied with L=4 quantization levels and a shared normal codebook.

$$C=\{e_{k}{\}}_{k=1}^{K},\;e_{k}\in R^{512},\;K=2048.$$ (3)

The same codebook is reused at each residual level. For the i-th token, residual quantization starts from $r_{i}^{\left(0\right)}=f_{i}$. At level l, the nearest codebook vector is selected and the residual is updated:

$$k_{i}^{\left(l\right)}=\arg\;\min_{k}{\left\|r_{i}^{\left(l-1\right)}-e_{k}\right\|}_{2}^{2},\;q_{i}^{\left(l\right)}=e_{k_{i}^{\left(l\right)}},\;r_{i}^{\left(l\right)}=r_{i}^{\left(l-1\right)}-q_{i}^{\left(l\right)}.$$ (4)

After all residual levels, the final quantized token is

$$q_{i}=\sum_{l=1}^{L}q_{i}^{\left(l\right)}.$$ (5)

The full quantized latent map is denoted as

$$Q=\{q_{i}{\}}_{i=1}^{N},\;Q\in R^{512\times 16\times 16}.$$ (6)

This residual design approximates the continuous token progressively. The first code captures the largest component of the token, while later levels encode the remaining residual information. Therefore, the final quantized token can represent normal CT latent features more accurately than a single-code approximation.

The decoder reconstructs the input image from the quantized latent map:

$$\hat{x}=D_{\mathrm{rec}}(Q).$$ (7)

During this normal reconstruction warm-up, the encoder, residual quantizer, and decoder are trained using only normal CT images. The training objective contains a reconstruction term and a commitment term:

$$L_{\mathrm{stage}1}=L_{\mathrm{rec}}+\beta L_{\mathrm{commit}},$$ (8)

where

$$L_{\mathrm{rec}}={\left\|x-\hat{x}\right\|}_{2}^{2}.$$ (9)

The reconstruction loss encourages the decoder to recover the input normal CT slice from the quantized latent map. However, reconstruction supervision alone is not sufficient for stable vector quantization, because the encoder may produce continuous features that drift away from the discrete codebook space. If this happens, the quantized representation may become unstable and the learned codebook may fail to serve as a compact normal feature repository.

To address this issue, a commitment loss is introduced to encourage the encoder output to remain close to its quantized approximation. In residual quantization, each token is approximated progressively across multiple residual levels. Therefore, the commitment loss is applied over all tokens and all residual levels:

$$L_{\mathrm{commit}}=\frac{1}{NL}\sum_{i=1}^{N}\sum_{l=1}^{L}{\left\|r_{i}^{\left(l-1\right)}-\mathrm{sg}\;\left(q_{i}^{\left(l\right)}\right)\right\|}_{2}^{2},$$ (10)

where N is the number of latent tokens, L is the number of residual quantization levels, $r_{i}^{\left(l-1\right)}$ is the residual feature before the l-th quantization step, $q_{i}^{\left(l\right)}$ is the selected code vector at that level, and sg(⋅) denotes the stop-gradient operation.

The stop-gradient operation prevents the commitment term from directly updating the selected code vector through this loss. Instead, it mainly pulls the encoder-side residual feature toward the selected quantized code, while the codebook itself is updated by exponential moving average. In this way, the encoder learns to produce features that can be consistently represented by the normal codebook, and the codebook gradually becomes a stable collection of normal latent prototypes. The coefficient β is the weight assigned to the commitment term relative to the reconstruction term and was held fixed throughout Stage I. A larger value enforces stronger encoder–codebook alignment, whereas a smaller value permits greater flexibility in the continuous representation.

After Stage I, the encoder learns CT-adapted normal latent features, the residual quantizer provides a discrete normal codebook space, and the decoder learns to reconstruct normal lung CT slices from the quantized latent map. These learned normal representations are then used by the reconstruction branch, the distance branch, and the transformer-based latent anomaly synthesis branch in the subsequent stages. The encoder representation, residual quantization procedure, reconstruction process, and Stage I training objective are defined in Equations 1–10.

### 3.3 Quantization-distance branch

As shown in Fig. 3, the distance branch is introduced to measure whether a local latent token can be well represented by the learned normal codebook. This branch is motivated by a limitation of reconstruction-only anomaly detection. In reconstruction-based scoring, the final residual is computed after decoding, and a sufficiently expressive decoder may partially reconstruct abnormal regions even when their latent features deviate from the learned normal representation. In such cases, the pixel-level reconstruction residual may underestimate the abnormality. The quantization-distance branch provides a complementary cue by measuring the mismatch before decoding, directly in the latent token space. Specifically, it compares each continuous encoder token with its quantized approximation from the normal codebook, thereby indicating how well the local feature can be represented by the learned normal latent prototypes. This cue is not intended to detect abnormalities that are fully represented by the normal codebook. Instead, it provides a complementary signal to the reconstruction residual by capturing latent-space deviations that may be weakened after decoder reconstruction.

**Figure 3:**
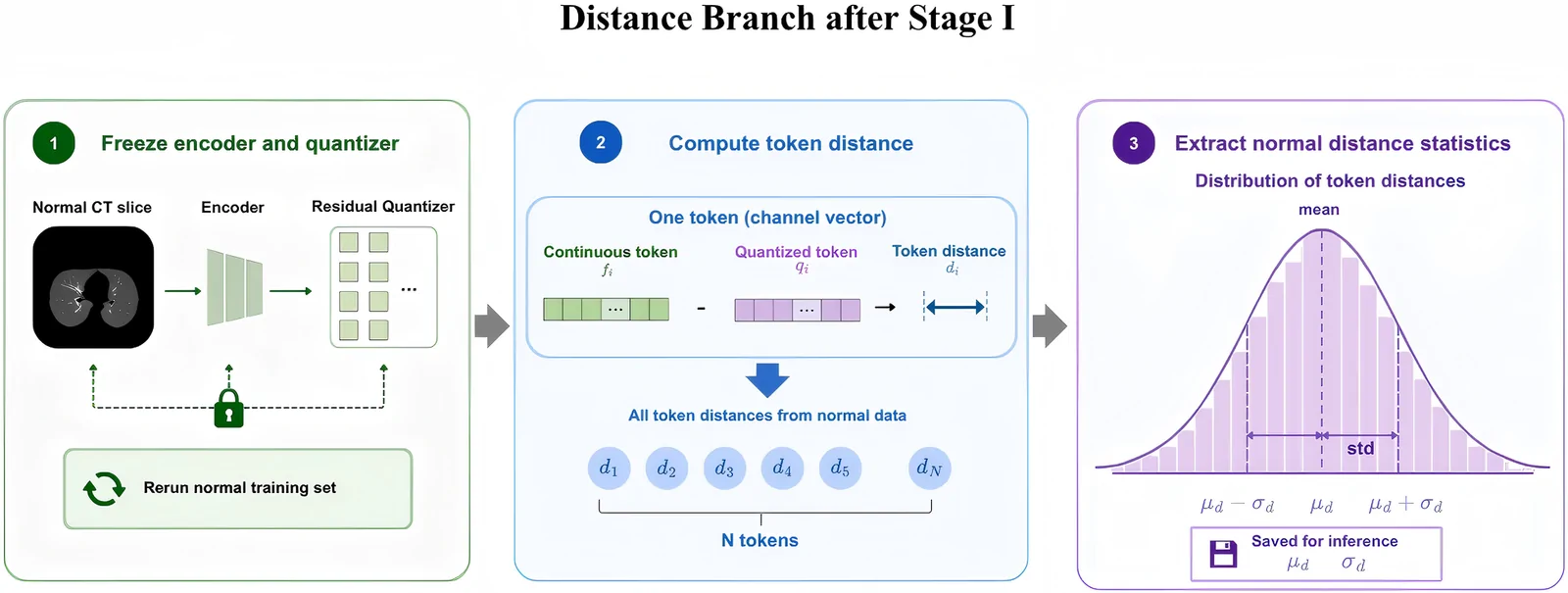
Quantization-distance branch after the normal reconstruction stage. The frozen encoder and residual quantizer are used to compute token-wise distances between continuous encoder tokens and quantized tokens. Normal distance statistics are saved and used for inference-time calibration.

After the reconstruction branch is trained, the encoder and quantizer are frozen. The normal training set is passed once through the frozen encoder and quantizer in inference mode to obtain the reference distribution of quantization distances. For each latent token, the distance between the continuous encoder token $f_{i}$ and its quantized token $q_{i}$ is computed as

$$d_{i}=\sqrt{\frac{1}{C}{\left\|f_{i}-q_{i}\right\|}_{2}^{2}},\;C=512.$$ (11)

If a token is well represented by the normal codebook, this distance should be small. The mean and standard deviation over normal training tokens are used as reference statistics:

$$\mu_{d}=\mathrm{Mean}\;(d_{i}),\;\sigma_{d}=\mathrm{Std}\;(d_{i}).$$ (12)

During inference, the quantization distance of each test token is normalized using these reference statistics:

$$z_{i}=\max\left(\frac{d_{i}-\mu_{d}}{\sigma_{d}+\epsilon },0\right).$$ (13)

Only distances larger than the normal range are retained. The token-level distance response is then upsampled and normalized to the input image resolution:

$$M_{\mathrm{dist}}=\frac{\mathrm{Up}\;(\{z_{i}{\}}_{i=1}^{N})}{\max(\mathrm{Up}\;(\{z_{i}{\}}_{i=1}^{N}))+\epsilon }.$$ (14)

The resulting map $M_{\mathrm{dist}}$ is the final output of the distance branch. It should be interpreted as a calibrated token-level normality deviation map rather than as the output of an additional learned classifier. A larger value in $M_{\mathrm{dist}}$ indicates that the corresponding encoder token is less well approximated by the learned normal codebook prototypes after normalization with normal training statistics. Since this response is computed before image decoding, it provides a latent-space cue that is different from the pixel-level reconstruction residual. In the final inference stage, $M_{\mathrm{dist}}$ is used as an enhancement factor to strengthen regions that already receive support from the reconstruction and transformer branches. The quantization-distance computation and calibration procedure are defined in Equations 11–14.

### 3.4 Transformer branch: latent anomaly synthesis and token discrimination

As illustrated in Fig. 4, the transformer branch is designed to learn token-level contextual abnormality through latent anomaly synthesis and token discrimination. Since lesion-level annotations are unavailable, direct supervised training is not possible. Therefore, a self-supervised token discrimination task is constructed by perturbing selected latent tokens within the learned codebook space.

**Figure 4:**
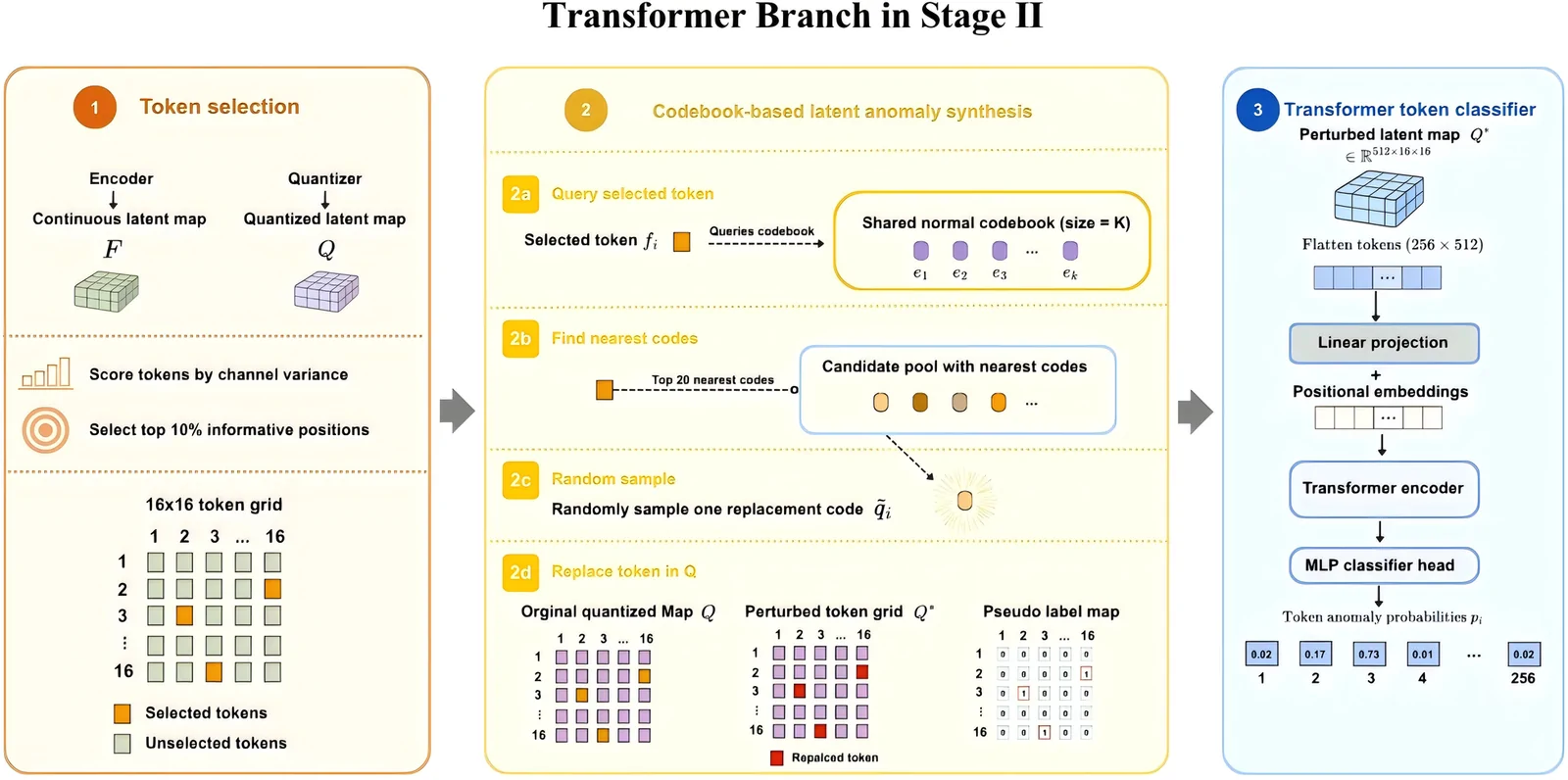
Transformer branch in Stage II. Selected latent tokens are replaced by sampled codebook candidates to construct a perturbed latent map. The transformer token classifier learns to distinguish replaced tokens from unchanged tokens.

#### 3.4.1 Latent token selection

The first step is to select informative token locations for perturbation. Instead of perturbing arbitrary positions, tokens with stronger local feature variation are selected. For each continuous encoder token $f_{i}$, the channel mean is

$${\bar{\;f}}_{i}=\frac{1}{C}\sum_{c=1}^{C}f_{i,c}.$$ (15)

The channel-wise variance score is

$$V_{i}=\frac{1}{C}\sum_{c=1}^{C}(f_{i,c}-{\bar{\;f}}_{i}{)}^{2}.$$ (16)

Tokens with larger variance are more likely to contain informative local structures. Therefore, the top 10% token positions are selected:

$$S=\mathrm{TopK}\;\left(\{V_{i}{\}}_{i=1}^{N},\left\lfloor 0.1N\right\rfloor \right).$$ (17)

Since N=256, approximately 25 positions are selected for each image.

#### 3.4.2 Codebook-based latent anomaly synthesis

For each selected token i∈S, the continuous token $f_{i}$ is used to query the shared normal codebook, and its 20 nearest codebook vectors are retrieved:

$$N_{i}=\mathrm{KNN}\;(f_{i},C,20).$$ (18)

Let $\nu_{i}(j)$ denote the codebook index of the j-th nearest candidate in $N_{i}$. A candidate index $j_{i}$ is sampled uniformly from {1,…,20}, and the replacement vector is defined as ${\tilde{q}}_{i}=e_{\nu_{i}(j_{i})}$. The perturbed token is therefore

$$q_{i}^{*}=\left\{ \begin{array}{cc} {\tilde{q}}_{i}, & i\in S,\\ q_{i}, & i\notin S. \end{array}\right.$$ (19)

This gives the perturbed latent map $Q^{*}=\{q_{i}^{*}{\}}_{i=1}^{N}$, where the token index i is preserved before and after replacement. The pseudo label is defined as

$$y_{i}=\left\{ \begin{array}{cc} 1, & i\in S,\\ 0, & i\notin S. \end{array}\right.$$ (20)

This synthesis process is different from adding random pixel noise. The replacement token still comes from the learned normal codebook, but it may become inconsistent with the local and global context after being inserted into another position. Therefore, the perturbation acts as a controlled latent disruption. The purpose is not to imitate a specific pulmonary disease, but to help the transformer learn what normal latent consistency should look like.

#### 3.4.3 Token discrimination

The perturbed latent map $Q^{*}$ is flattened into a token sequence and fed into the transformer token classifier. Positional embeddings are added to preserve spatial information. The transformer encoder and MLP head output a token-level anomaly probability:

$$p_{i}=T_{\psi }(Q^{*}{)}_{i},\;p_{i}\in [0,1].$$ (21)

The transformer is trained to distinguish replaced tokens from unchanged tokens according to the pseudo labels $y_{i}$. In this way, the branch learns token-level contextual inconsistency in the latent space.

#### 3.4.4 Reliability weighting with auxiliary reconstruction

Not all synthetic replacements are equally informative. Some replacements may create visible reconstruction changes, while others may be too weak. To reduce the influence of unreliable perturbations, an auxiliary reconstruction branch is introduced to estimate token reliability.

The perturbed latent map is decoded as

$$x^{*}=D_{\mathrm{aux}}(Q^{*}).$$ (22)

A pooled residual map is computed between the original image and the auxiliary reconstruction:

$$R_{\mathrm{re}}=\left|\mathrm{AvgPool}\;(x)-\mathrm{AvgPool}\;(x^{*})\right|.$$ (23)

This residual map is divided into token-aligned patches. For the patch corresponding to token i, the patch mean and variance are computed and combined into a reliability weight:

$$w_{i}=\mathrm{Norm}\;\left(\mathrm{Mean}\;(R_{i})\right)+\mathrm{Norm}\;\left(\mathrm{Var}\;(R_{i})\right).$$ (24)

Here, Norm(⋅) denotes token-wise min–max normalization within each slice. For token values $\{a_{i}{\}}_{i=1}^{N}$, it is defined as

$$\mathrm{Norm}\;(a_{i})=\frac{a_{i}-\min_{j}a_{j}}{\max_{j}a_{j}-\min_{j}a_{j}+\epsilon },$$ (25)

where ϵ is a small positive constant used for numerical stability. The same normalization is applied separately to the patch-mean and patch-variance responses. A larger $w_{i}$ indicates that the perturbation produces a stronger and more reliable reconstruction response.

The transformer classification loss is then written as

$$L_{\mathrm{cls}}=\frac{1}{N}\sum_{i=1}^{N}\gamma_{i}\mathrm{CE}\;(p_{i},y_{i}),\;\gamma_{i}=\left\{ \begin{array}{cc} w_{i}, & i\in S,\\ 1, & i\notin S. \end{array}\right.$$ (26)

The auxiliary reconstruction loss is

$$L_{\mathrm{rec}}^{\left(2\right)}={\left\|x-D_{\mathrm{aux}}(Q^{*})\right\|}_{2}^{2}.$$ (27)

The overall objective for this branch is

$$L_{\mathrm{stage}2}=L_{\mathrm{cls}}+\lambda_{\mathrm{rec}}L_{\mathrm{rec}}^{\left(2\right)}.$$ (28)

Here, $\lambda_{\mathrm{rec}}$ weights the auxiliary reconstruction loss relative to the token-classification loss and was held fixed throughout Stage II. If $\lambda_{\mathrm{rec}}$ is too small, the auxiliary decoder may provide weak reliability information and the transformer may overfit noisy pseudo labels. If it is too large, reconstruction may dominate the objective and weaken token discrimination. The latent token selection, codebook-based perturbation, token discrimination, reliability weighting, and Stage II objective are defined in Equations 15–28.

### 3.5 Inference and final anomaly scoring

During inference, no latent replacement is performed. A test image x is passed through the frozen encoder and residual quantizer to obtain the continuous latent tokens $F=\{f_{i}{\}}_{i=1}^{N}$ and the quantized latent tokens $Q=\{q_{i}{\}}_{i=1}^{N}$. The final anomaly score is computed by combining three anomaly maps: a reconstruction residual map, a transformer token response map, and a quantization-distance map.

The first cue is the reconstruction residual map. The quantized latent representation Q is decoded back to the image space:

$${\hat{x}}_{\mathrm{rec}}=D_{\mathrm{rec}}(Q).$$ (29)

The reconstruction residual map is then computed as the absolute pixel-wise difference between the input image and its reconstruction:

$$M_{\mathrm{rec}}=\left|x-{\hat{x}}_{\mathrm{rec}}\right|.$$ (30)

This map captures appearance-level discrepancy in the image space. Regions that cannot be faithfully reconstructed from the learned normal quantized representation tend to produce larger residual responses.

The second cue is the transformer token response map. The original quantized latent map Q is fed into the trained transformer token classifier. The transformer predicts a token-level abnormality probability for each latent position, and the resulting token responses are upsampled to the input image resolution:

$$M_{\mathrm{cls}}=\mathrm{Up}\;\left(\{p_{i}{\}}_{i=1}^{N}\right).$$ (31)

This map reflects whether each latent token appears contextually inconsistent with the learned normal token structure.

The third cue is the quantization-distance map $M_{\mathrm{dist}}$, defined in Section 3.3. Different from the reconstruction residual map, which is computed after decoding in the image space, the distance map is computed before decoding in the latent token space. It measures how well each continuous encoder token can be represented by the learned normal codebook.

The reconstruction and transformer maps are first combined by element-wise multiplication:

$$A_{\mathrm{rt}}=M_{\mathrm{cls}}⊙M_{\mathrm{rec}},$$ (32)

where ⊙ denotes element-wise multiplication. This operation keeps regions that are supported by both image-space reconstruction discrepancy and token-level transformer response. In other words, the reconstruction map provides fine spatial detail, while the transformer map suppresses residual responses that are less consistent with abnormal token-level context.

The distance map is then used as an enhancement factor:

$$A_{\mathrm{fuse}}=A_{\mathrm{rt}}⊙\left(1+\lambda_{\mathrm{dist}}M_{\mathrm{dist}}\right),$$ (33)

where $\lambda_{\mathrm{dist}}\in [0,1]$ controls the contribution of the distance cue. Unless otherwise specified, $\lambda_{\mathrm{dist}}=0.7$ is used in the main experiments. This design allows the distance branch to strengthen regions that are not only suspicious in the reconstruction-transformer response but also deviate from the learned normal codebook space.

Finally, the slice-level anomaly score is obtained by averaging the positive responses in the fused anomaly map:

$$\Omega_{A}=\{u|A_{\mathrm{fuse}}(u)>0\},\;s(x)=\frac{\sum_{u\in \Omega_{A}}A_{\mathrm{fuse}}(u)}{|\Omega_{A}|+1}.$$ (34)

The output of the model therefore includes both a scalar slice-level anomaly score and a spatial anomaly heatmap. The inference-time reconstruction, transformer response, multi-cue fusion, and slice-level anomaly scoring procedures are defined in Equations 29–34.

### 3.6 Datasets and evaluation cohorts

Three publicly available datasets were combined for the primary pulmonary CT anomaly-detection experiments. The resulting benchmark contained normal controls and five abnormal categories: COVID-19, community-acquired pneumonia (CAP), adenocarcinoma, nontuberculous mycobacterial pulmonary disease (NTM), and tuberculosis (TB). After preprocessing, the primary benchmark contained 490 cases and 32,338 retained 2D slices. The composition of the primary pulmonary CT datasets is summarized in Table 1.

**Table 1:** Primary pulmonary CT datasets after preprocessing and slice selection.

Dataset	Class	Cases	Retained slices
COVID-CT-MD	Normal	76	7,956
COVID-CT-MD	COVID-19	169	8,534
COVID-CT-MD	CAP	60	3,871
Lung-PET-CT-Dx	Adenocarcinoma	71	1,454
Integrated mycobacterial CT	NTM	64	7,510
Integrated mycobacterial CT	TB	50	3,013
Total	–	490	32,338

The slice counts correspond to the lesion-enriched slice-level cohort.

#### 3.6.1 COVID-19, normal, and CAP cohort

The COVID-CT-MD dataset (25) contains 305 chest CT studies, including 169 COVID-19 cases, 60 CAP cases, and 76 normal cases. After preprocessing and slice screening, 7,956 normal, 8,534 COVID-19, and 3,871 CAP slices were retained.

#### 3.6.2 Lung cancer cohort

The Lung-PET-CT-Dx dataset (26) contains retrospectively collected CT and PET/CT studies from subjects with suspected lung cancer. Only CT images from 71 adenocarcinoma cases were used, resulting in 1,454 retained slices. The available bounding-box annotations were not used for training.

#### 3.6.3 NTM and TB cohort

The integrated mycobacterial CT dataset (27) includes NTM and TB cases acquired using thin-slice chest CT protocols. After preprocessing, 64 NTM cases with 7,510 slices and 50 TB cases with 3,013 slices were retained.

#### 3.6.4 Slice-level and patient-level cohorts

Two complementary evaluation protocols were used. For the original slice-level analysis, abnormal slices were manually screened during dataset preparation. Only slices with a visually apparent pulmonary abnormality and adequate lung-region coverage were retained; slices from abnormal volumes that appeared visually normal, contained no evident lesion, or showed incomplete lung regions were excluded. Normal slices were selected from normal CT volumes using the same lung-coverage criterion. Available lesion annotations were used as supporting information where present, but a uniform lesion-annotation standard was not available across all three source datasets. Consequently, the slice-level cohort should be interpreted as a lesion-enriched evaluation set rather than as an unfiltered collection in which every slice inherited the patient diagnosis.

For patient-level evaluation, this lesion-enrichment step was not applied. All valid lung-containing slices from each test CT volume were retained, including slices that appeared visually normal in patients with localized disease. Slice scores were subsequently aggregated into one patient-level score as described in Section 3.8. This protocol avoids assigning a binary abnormal label to every individual slice from an abnormal patient.

To prevent patient leakage, splitting was performed at the case level. Of the 76 normal cases, 60 were assigned to training, 7 to validation/calibration, and 9 to the held-out test set, corresponding to the predefined 8:1:1 splitting rule. All abnormal cases were reserved for testing and were not used to train the model or determine decision thresholds. The limited numbers of normal validation/calibration and test patients should be considered when interpreting threshold calibration, specificity, balanced accuracy, and patient-level generalizability.

#### 3.6.5 External LIDC-IDRI localization cohort

Quantitative localization was additionally evaluated using the LIDC-IDRI database (28). Only CT cases containing at least one physical nodule cluster annotated by three or more radiologists were eligible. Individual annotations referring to the same physical nodule were first grouped using the annotation clustering provided by pylidc. For each eligible nodule cluster, a three-dimensional consensus mask was then generated using pylidc.utils.consensus with a consensus level of 0.5. Thus, for a cluster with $R_{c}$ radiologist annotations, a voxel v was included in the cluster-level reference mask when

$$M_{c}(v)=I\left[\frac{1}{R_{c}}\sum_{r=1}^{R_{c}}M_{c,r}(v)\geq 0.5\right],$$ (35)

where $M_{c,r}(v)$ is the binary mask assigned to voxel v by radiologist r for nodule cluster c. This corresponds to a 50% consensus rule rather than a simple union or intersection of the individual annotations. When a patient contained more than one eligible nodule cluster, the cluster-level consensus masks were combined by voxel-wise union to form the final patient-level lesion volume,

$$M_{\mathrm{ref}}=\underset{c\in C_{\mathrm{eligible}}}{⋃}M_{c}.$$ (36)

Only slices with a non-empty final consensus lesion mask were retained for localization evaluation. Binary lesion masks were resized from the source image resolution to 128×128 using nearest-neighbour interpolation to preserve discrete mask labels. After this consensus-based filtering, 100 cases were included. No LIDC-IDRI case was used for model training, model or hyperparameter selection, checkpoint selection, score calibration, or threshold selection; the cohort was used only for the independent localization evaluation. Because many annotated nodules are small and low contrast, this evaluation provides a challenging slice-level test of anomaly localization. The consensus-mask construction is defined in Equations 35–36.

### 3.7 Implementation details

#### 3.7.1 Preprocessing and input preparation

Before lung-region extraction, CT intensities were windowed using a lung window with a window level of −600 HU and a window width of 1,500 HU, corresponding to an intensity range of −1350 to 150 HU. Values outside this range were clipped, and the windowed intensities were linearly mapped to 8-bit grayscale values in [0,255]. Binary lung masks were then generated using the publicly available lungmask toolkit with the pretrained U-Net R231 model (29). The segmentation model was not retrained. Pixels outside the lung masks were set to zero, and slices with inadequate lung-region coverage were excluded. The remaining single-channel lung-region images were resized to 128×128.

#### 3.7.2 Training details

All experiments were implemented in PyTorch and conducted on a single NVIDIA GeForce RTX 4090 GPU with 16 GB memory.

The framework was optimized in two stages. In Stage I, a reconstruction-based warm-up was performed using only normal training images. The encoder was a modified ImageNet-pretrained ResNet18 adapted for grayscale input. Given an input image of size 128×128, the encoder produced a latent representation of size 16×16×512. This latent feature map was discretized using four residual quantization levels with a shared codebook containing 2,048 embeddings. A reconstruction decoder was jointly trained with the encoder and quantizer for 50 epochs using AdamW with a learning rate of $1\times 10^{-4}$, a batch size of 16, and optimizer betas of (0.5,0.9).

In Stage II, the pretrained encoder and quantization module were frozen, while the decoder-related modules and transformer branch were optimized. The transformer operated on 256 latent tokens of dimension 512 and contained 12 layers, 16 attention heads, a head dimension of 32, a hidden dimension of 2,048, and dropout of 0.1. The top 10% of latent tokens were selected for codebook-based perturbation. Stage II was trained for 20 epochs using Adam with a learning rate of $1\times 10^{-4}$ and a batch size of 16.

#### 3.7.3 Inference setting

The fused anomaly scoring strategy in Section 3.5 was used. Unless otherwise specified, $\lambda_{\mathrm{dist}}=0.7$. Its influence was evaluated from 0.1 to 1.0 in the ablation study.

### 3.8 Evaluation protocol and metrics

#### 3.8.1 Slice-level scoring and threshold calibration

For the lesion-enriched slice-level cohort, each retained CT slice was treated as one observation. A Gaussian reference model was fitted using anomaly scores from normal training/calibration slices. For a slice score s, the calibrated response was

$$z_{s}=\frac{s-\mu_{s}}{\sigma_{s}+\epsilon },\;p_{s}=\mathrm{sigmoid}\;(z_{s}),$$ (37)

where $\mu_{s}$ and $\sigma_{s}$ are estimated only from normal calibration scores. A slice was classified as abnormal when $p_{s}\geq 0.5$. No abnormal test slice was used to determine this threshold. The slice-level calibration rule is defined in Equation 37.

#### 3.8.2 Patient-level aggregation and threshold calibration

For patient j with $n_{j}$ valid slice scores, the scores were sorted in descending order, $s_{\;j,(1)}\geq \cdots \geq s_{\;j,(n_{j})}$. The patient-level score was the mean of the highest 10% of slice scores:

$$S_{j}=\frac{1}{k_{j}}\sum_{r=1}^{k_{j}}s_{\;j,(r)},\;k_{j}=\max\left(1,\left\lceil 0.1n_{j}\right\rceil \right).$$ (38)

The 10% aggregation fraction was specified before examining the patient-level test results and was not selected or tuned using test-set AUROC or any other test-set metric. A sensitivity analysis using mean, maximum, and top-10% aggregation was subsequently performed to assess whether the comparative conclusion depended on this prespecified choice. Thus, each patient contributed exactly one independent observation. Let $V_{0}$ denote the set of normal validation/calibration patients and let $N_{\mathrm{val}}=|V_{0}|$. The patient-level decision threshold was defined as

$$\tau_{\mathrm{pat}}=\frac{1}{N_{\mathrm{val}}}\sum_{\;j\in V_{0}}S_{j},$$ (39)

and the predicted patient label was

$${\hat{y}}_{j}=\left\{ \begin{array}{cc} 1, & S_{j}\geq \tau_{\mathrm{pat}},\\ 0, & S_{j}<\tau_{\mathrm{pat}}. \end{array}\right.$$ (40)

Neither abnormal test patients nor held-out normal test patients influenced threshold selection. The same calibration and aggregation procedure was applied separately to the proposed method and every baseline. The patient-level aggregation and thresholding procedures are defined in Equations 38–40.

#### 3.8.3 Metrics and confidence intervals

AUROC was the primary threshold-independent metric at both slice and patient levels. Recall/sensitivity, specificity, precision, F1-score, and conventional accuracy were computed under the fixed calibrated threshold. Because the patient-level test cohort was strongly imbalanced, balanced accuracy was emphasized in the main patient-level comparison. Balanced accuracy gives equal weight to sensitivity and specificity, whereas conventional accuracy can be dominated by the majority class.

Patient-level uncertainty was estimated using a class-stratified bootstrap with 2,000 replicates. In each replicate, normal test patients were sampled with replacement to the original number of normal test patients, and abnormal test patients were sampled independently with replacement to the original number of abnormal test patients. The two samples were combined and AUROC was recalculated. The 2.5th and 97.5th percentiles of the bootstrap distribution formed the 95% confidence interval. The independently calibrated threshold was held fixed in every replicate; therefore, the bootstrap quantifies uncertainty due to patient sampling while keeping the model and decision rule unchanged. For the paired comparison between the proposed method and UTRAD, the same resampled patient indices were applied to both methods within each bootstrap replicate, and the AUROC difference was calculated as $\Delta \mathrm{AUROC}={\mathrm{AUROC}}_{\mathrm{proposed}}-{\mathrm{AUROC}}_{\mathrm{UTRAD}}$. The percentile interval of these paired AUROC differences was used as the 95% confidence interval for the between-method difference.

For quantitative localization on LIDC-IDRI, the reference labels were the final 50%-consensus lesion masks described above, after voxel-wise union across multiple eligible nodules within the same patient when applicable. P-AUROC was computed by pooling the pixel-level anomaly scores and reference-mask labels from all lesion-containing slices across the 100-case cohort and calculating a single whole-cohort pixel-level AUROC. The reported P-AUROC was therefore a global pooled-pixel measure rather than an average of separately calculated per-case AUROCs.

### 3.9 Comparison methods

The proposed method was compared with five representative anomaly detection baselines covering different methodological families, including pretrained feature-based modeling, memory-bank retrieval, self-supervised one-class learning, normalizing-flow-based density modeling, and reconstruction-based modeling.

#### 3.9.1 SimpleNet

SimpleNet (23) is a pretrained feature-based anomaly detection framework. It extracts local features using a pretrained backbone, adapts them with a lightweight feature adapter, generates synthetic anomaly features in feature space, and trains a discriminator to distinguish normal from anomalous features.

#### 3.9.2 PatchCore

PatchCore (19) is a memory-bank-based anomaly detection method. It constructs a representative memory bank of normal patch features and detects anomalies by measuring the nearest-neighbor distance between test-time features and stored normal prototypes.

#### 3.9.3 CutPaste

CutPaste (22) is a self-supervised one-class anomaly detection method. It learns discriminative representations by training a model to distinguish original normal images from cut-and-paste augmented images, and then uses the learned representation space for anomaly scoring.

#### 3.9.4 CS-Flow

CS-Flow (21) is a normalizing-flow-based anomaly detection approach. It models the distribution of normal multi-scale features using fully convolutional cross-scale flows and identifies anomalies according to low-likelihood regions under the learned normal density.

#### 3.9.5 UTRAD

UTRAD (30) is a reconstruction-based anomaly detection framework. It represents deep pretrained features as token sequences and employs a U-Transformer autoencoder to reconstruct normal feature distributions, where reconstruction discrepancies are used for anomaly detection and localization.

For fair comparison, all baseline methods were evaluated using the same slice-level protocol; the baselines included in the patient-level analysis were additionally evaluated using the same patient aggregation and calibration procedure.

## 4 Results

### 4.1 Slice-level anomaly detection

Table 2 summarizes the 2D slice-level anomaly detection results of the proposed method and five representative baseline methods. As shown in the table, the proposed method achieved the highest observed values across all four evaluation metrics, yielding an AUROC of **0.9218**, a Recall of **0.9611**, an F1-score of **0.9703**, and an Accuracy of **0.9435**. These values indicate favorable observed anomaly-discrimination performance among the compared methods, without implying a formal between-method significance test.

**Table 2:** 2D slice-level anomaly-detection results.

Method	AUROC	Recall	F1-score	Accuracy
SimpleNet	0.8853	0.9461	0.9594	0.9232
PatchCore	0.8790	0.9190	0.9460	0.8990
CutPaste	0.8561	0.9428	0.9463	0.9355
CS-Flow	0.8918	0.8919	0.9355	0.8821
UTRAD	0.9046	0.9443	0.9625	0.9294
Ours	**0.9218**	**0.9611**	**0.9703**	**0.9435**

The highest observed value in each column is shown in bold.

Among the compared baselines, UTRAD achieves the highest AUROC and F1-score, suggesting that reconstruction-based modeling remains a strong strategy for chest CT anomaly detection. SimpleNet obtains the highest Recall among the baselines, while CutPaste shows relatively high Accuracy. However, no baseline method consistently achieved the highest observed value across all metrics. The proposed method had the highest observed value on all four evaluation metrics, with an AUROC of 0.9218, Recall of 0.9611, F1-score of 0.9703, and Accuracy of 0.9435.

The comparison also shows that different baseline families have different strengths. Feature-based methods such as SimpleNet and PatchCore obtain competitive Recall, but their AUROC values remain lower than that of the proposed method, indicating weaker threshold-independent discrimination. CutPaste achieves a relatively high Accuracy under the unified thresholding rule, but it obtains the lowest AUROC among the compared methods, suggesting that its overall ranking ability is limited in this CT anomaly detection setting. CS-Flow provides competitive AUROC compared with several baselines, but its threshold-dependent metrics are lower than those of the strongest methods. UTRAD gives the strongest baseline AUROC and F1-score, while the proposed method yielded higher observed values for both metrics.

Overall, the proposed framework showed favorable observed performance across the evaluated metrics. Relative to the strongest baseline values, the observed AUROC was 0.9218 vs. 0.9046, Recall was 0.9611 vs. 0.9461, F1-score was 0.9703 vs. 0.9625, and Accuracy was 0.9435 vs. 0.9355. These descriptive differences are consistent with the intended complementarity of pixel-level reconstruction residuals, transformer-based token discrimination, and latent quantization-distance information, but are not presented as formal between-method significance tests.

### 4.2 Patient-level anomaly detection

Table 3 reports patient-level results obtained after the prespecified top-10%-mean aggregation. The proposed method achieved the highest observed AUROC and F1-score among the evaluated methods. Because abnormal patients substantially outnumbered held-out normal patients, balanced accuracy was emphasized over conventional accuracy: a classifier that predicts the majority abnormal class frequently can obtain a deceptively high conventional accuracy, whereas balanced accuracy weights sensitivity and specificity equally.

**Table 3:** Patient-level anomaly-detection performance using top-10%-mean slice-score aggregation.

Method	AUROC	Balanced accuracy	F1-score
Proposed method	** 0.918731±0.012298 **	** 0.752102±0.035414 **	** 0.929697±0.005168 **
UTRAD	0.884344±0.012596	0.739355±0.026738	0.903768±0.012951
PatchCore	0.855866±0.024457	0.740400±0.035689	0.867087±0.090786
SimpleNet	0.765053±0.010293	0.669280±0.016326	0.544764±0.047129
CS-Flow	0.740135±0.012952	0.685867±0.005436	0.712798±0.044749

Values are reported as mean ± standard deviation.

The patient-stratified bootstrap yielded a 95% AUROC confidence interval of 0.8786–0.9727 for the proposed method and 0.8266–0.9514 for UTRAD, the strongest patient-level baseline. A paired patient-level bootstrap was additionally performed using identical resampled patient indices for both methods. The observed AUROC difference was ΔAUROC=0.0344 in favor of the proposed method, and the paired 95% bootstrap confidence interval for the difference was −0.0501 to 0.1233. Because this interval included zero, the between-method difference was not statistically conclusive under the paired bootstrap analysis. Accordingly, the proposed method is described as achieving a higher observed patient-level AUROC than UTRAD rather than demonstrating statistically significant superiority.

#### 4.2.1 Sensitivity to patient-level aggregation

To examine whether the patient-level conclusion depended on the prespecified top-10% rule, AUROC was recalculated using three aggregation strategies: the mean of all valid slice scores, the maximum slice score, and the mean of the highest 10% of slice scores. The same aggregation rule was applied to every method within each comparison. Table 4 shows that the absolute AUROC values changed with the aggregation strategy, while the proposed method retained the highest observed AUROC under all three rules.

**Table 4:** Sensitivity of patient-level AUROC to the slice-to-patient aggregation rule.

Method	Mean	Maximum	Top-10% mean
Proposed method	** 0.865940±0.006411 **	** 0.882075±0.024017 **	** 0.918731±0.012298 **
UTRAD	0.840264±0.027448	0.867985±0.012735	0.884344±0.012596
PatchCore	0.681285±0.040495	0.786644±0.064160	0.855866±0.024457
SimpleNet	0.688480±0.097095	0.749777±0.022832	0.765053±0.010293
CS-Flow	0.640483±0.021595	0.695849±0.030561	0.740135±0.012952

Values are reported as mean ± standard deviation across repeated runs. The top-10% rule was the prespecified primary analysis; mean and maximum aggregation were included as sensitivity analyses.

The sensitivity analysis illustrates how the slice-to-patient aggregation rule can affect pulmonary CT anomaly detection. Averaging all slices can dilute focal abnormality signals because many slices in an abnormal volume may contain little or no visible disease, whereas maximum aggregation can be overly influenced by a single spuriously high-scoring slice or imaging artifact. The prespecified top-10% mean provides an intermediate strategy by emphasizing the most suspicious portion of the CT volume while averaging across multiple slices. Importantly, the proposed method retained the highest observed AUROC under mean, maximum, and top-10% aggregation, indicating that its comparative ranking was not dependent on the primary aggregation rule alone. The top-10% rule was retained as the primary analysis because it had been defined before examination of the patient-level test results rather than selected retrospectively from the sensitivity analysis.

### 4.3 Source-stratified patient-level analysis

Table 5 reports disease-wise patient-level performance for all five abnormal categories included in the source-stratified analysis. Within COVID-CT-MD, where normal, COVID-19, and CAP cases originate from the same source dataset, the proposed method achieved AUROCs of 0.8470 for COVID-19 and 0.9747 for CAP. Importantly, these comparisons reduce the possibility that discrimination is driven solely by dataset identity, since both the normal and abnormal cases were acquired within the same dataset. The method also maintained high patient-level discrimination for adenocarcinoma from Lung-PET-CT-Dx and for NTM and TB from the integrated mycobacterial CT dataset, with AUROCs of 0.9696, 0.9167, and 0.9481, respectively.

**Table 5:** Disease-wise patient-level results for the source-stratified analysis.

Source dataset	Abnormal category	AUROC	Balanced accuracy	F1-score
COVID-CT-MD	COVID-19	0.847030±0.026004	0.698444±0.037090	0.858654±0.011757
COVID-CT-MD	CAP	** 0.974691±0.006503 **	** 0.798148±0.032075 **	0.956058±0.004537
Lung-PET-CT-Dx	Adenocarcinoma	0.969559±0.003317	0.795408±0.033460	** 0.968913±0.006091 **
Integrated mycobacterial CT	NTM	0.916667±0.017099	0.736690±0.040570	0.889978±0.018202
Integrated mycobacterial CT	TB	0.948148±0.007143	0.771481±0.037849	0.922559±0.011664

Each abnormal category was compared with the held-out normal patient cohort. Values are mean ± standard deviation.

These results do not completely eliminate the possibility of dataset-source confounding, because matched normal controls are not available within every source dataset. However, the preservation of discrimination in the same-source COVID-CT-MD comparisons, together with consistently high performance across the other abnormal categories, provides no clear evidence that the proposed method relies exclusively on scanner, acquisition, or preprocessing-related differences rather than pulmonary abnormality patterns.

### 4.4 Quantitative anomaly localization on LIDC-IDRI

Quantitative localization performance on the external LIDC-IDRI cohort is reported in Table 6. The proposed method achieved a P-AUROC of 0.9155, the highest observed value among the evaluated methods (UTRAD 0.8307, PatchCore 0.7856, CS-Flow 0.6394, and SimpleNet 0.4012). The reference lesion labels were generated using the 50% radiologist-consensus procedure described in the dataset section, with voxel-wise union across multiple eligible nodules within a patient. P-AUROC was calculated once from the pooled pixel-level predictions and reference labels across all lesion-containing slices in the 100-case cohort, rather than by averaging per-case AUROCs. The experiment was performed only at the slice and pixel levels because the reference annotations describe individual nodules rather than a patient-level normal/abnormal classification task.

**Table 6:** Pixel-level anomaly localization on the 100-case LIDC-IDRI consensus cohort.

Method	P-AUROC
Proposed method	**0.9155**
UTRAD	0.8307
PatchCore	0.7856
CS-Flow	0.6394
SimpleNet	0.4012

Higher P-AUROC indicates better separation of annotated nodule pixels from other pixels. The highest observed P-AUROC is shown in bold.

The relatively low P-AUROC of SimpleNet (0.4012) may be related to the particularly challenging nature of the LIDC-IDRI localization setting, where many nodules are small, low-contrast, and locally similar to normal anatomical structures such as vessels or bronchi. SimpleNet primarily learns local feature-level anomaly discrimination through synthetic feature perturbations and a discriminator, but does not explicitly model the contextual relationships among neighboring latent regions. Consequently, a small nodule whose local appearance is not strongly separable from normal pulmonary structures may receive only a weak anomaly response, while visually similar normal structures may produce competing activations. In contrast, the proposed framework incorporates transformer-based contextual token discrimination, which evaluates whether a latent token is consistent with its surrounding normal context, and further combines this response with reconstruction discrepancy and quantization-distance information. This multi-cue design provides additional evidence for subtle abnormalities and may therefore improve the spatial ranking of small nodule pixels relative to surrounding normal tissue.The resulting P-AUROC on the LIDC-IDRI cohort therefore provides complementary quantitative evidence that the proposed multi-cue representation is able to localize subtle pulmonary abnormalities beyond the qualitative multi-disease evaluation.

### 4.5 Qualitative anomaly localization

The localization ability of the model was evaluated quantitatively on the external LIDC-IDRI nodule cohort (Section 4.4) and qualitatively on representative samples from the multi-disease cohort. Fine-grained lesion annotations were not used for training and were not uniformly available for the primary disease datasets; therefore, the qualitative analysis remains complementary to the external pixel-level evaluation.

Figure 5 presents the intermediate outputs of the proposed framework for three representative abnormal categories, namely CAP, NTM, and TB. From left to right, the figure shows the input CT slice, reconstructed image, fused anomaly heatmap, transformer response map, reconstruction residual map, and quantization-distance response map. Several observations can be made from this figure. First, the reconstructed images preserve the main anatomical structure of the lungs well, indicating that the reconstruction branch successfully models the dominant normal appearance. Second, the residual maps highlight suspicious regions but are relatively scattered and contain noticeable low-level noise, suggesting that reconstruction discrepancy alone is not sufficiently stable for precise localization. Third, the transformer probability maps provide semantically meaningful responses, but remain spatially coarse because they originate from the upsampled token grid. In contrast, the quantization-distance maps are visually cleaner and more concentrated, indicating that deviation from the learned normal feature manifold provides a more structured anomaly signal. After fusing these complementary cues according to Equation 33, the final heatmaps become more localized and more consistent with the suspicious lesion regions. This result suggests that the three branches are complementary rather than redundant.

**Figure 5:**
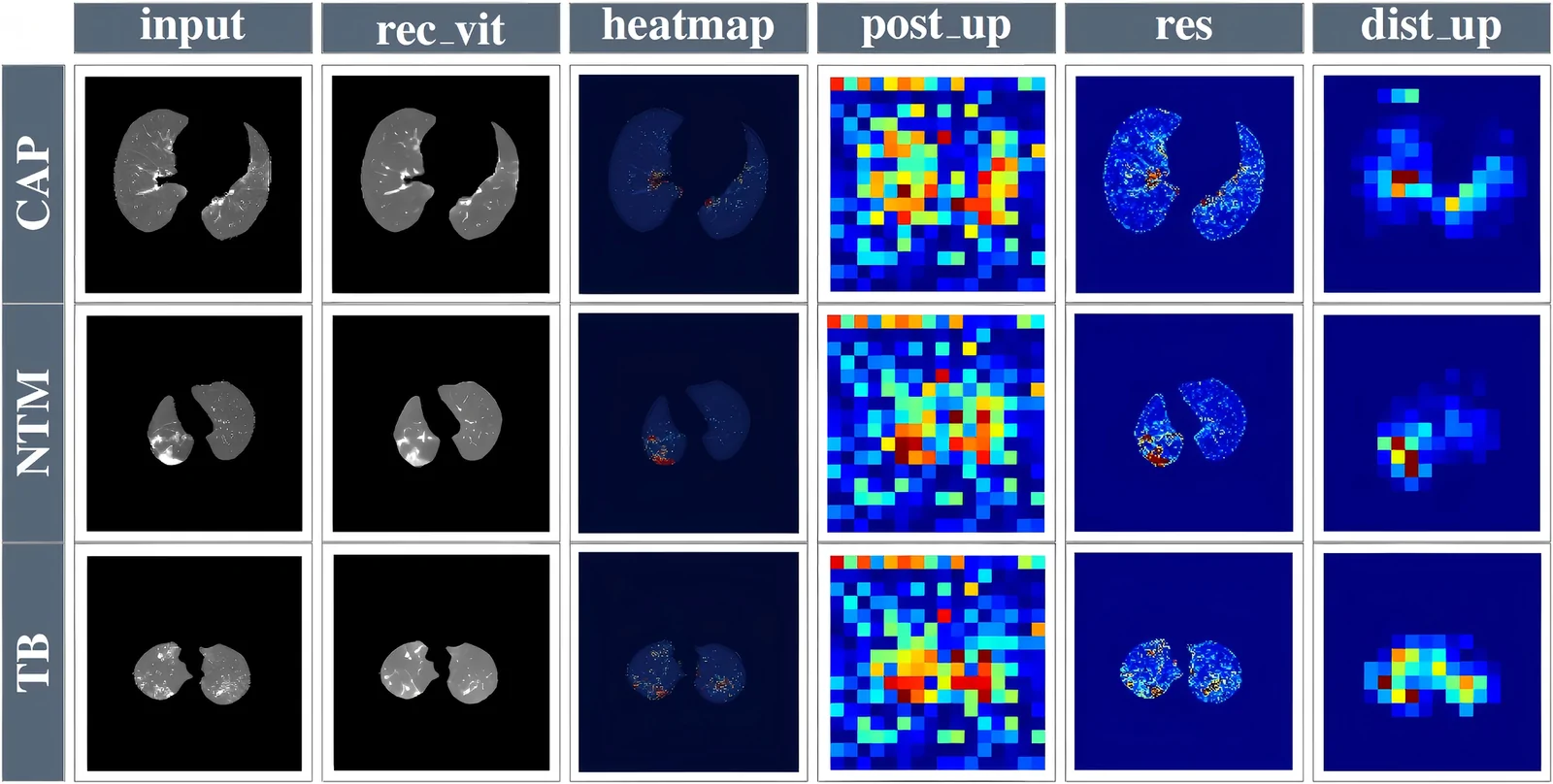
Intermediate outputs for three representative abnormal categories. From left to right, the columns show the input CT slice, reconstructed image, fused anomaly heatmap, transformer response map, reconstruction residual map, and quantization-distance response map. From top to bottom, the rows correspond to CAP, NTM, and TB.

Figure 6 compares the qualitative anomaly localization results of the proposed method with five representative baseline methods, including SimpleNet, PatchCore, CutPaste, CS-Flow and UTRAD. Among the compared baselines, CS-Flow shows the weakest localization performance in these examples. Its responses often fail to cover the visually abnormal regions and may instead activate unrelated areas, indicating limited spatial reliability for pulmonary CT lesion localization. CutPaste performs slightly better than CS-Flow in some samples, as it can roughly respond to part of the abnormal region. However, its localization remains unstable, and obvious mislocalization can still be observed, such as in the adenocarcinoma sample. This suggests that CutPaste-style image-space patch augmentation may not always provide localization cues that are well aligned with subtle or irregular CT lesion morphology.

**Figure 6:**
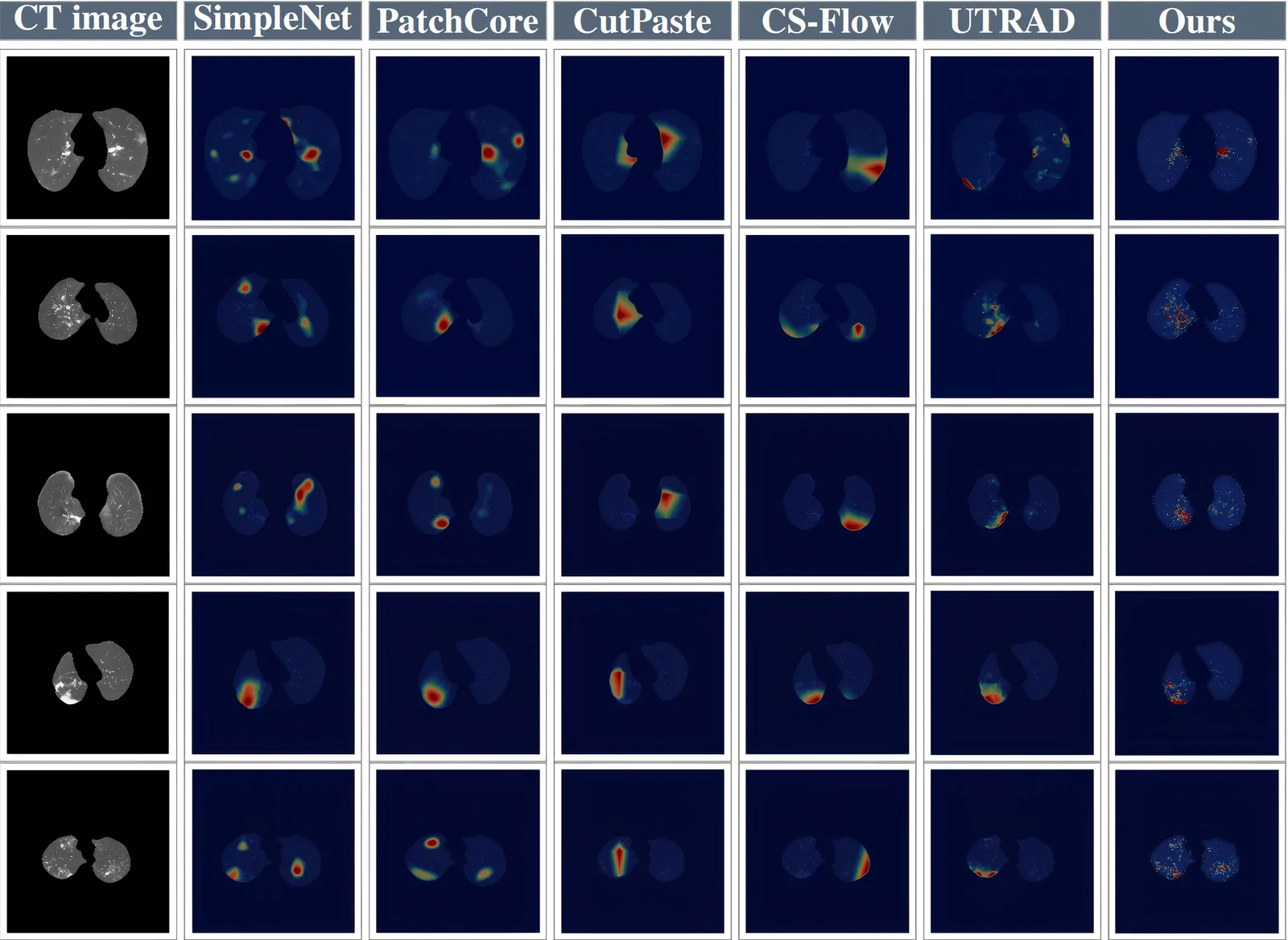
Qualitative comparison of anomaly localization results for five representative abnormal categories. From top to bottom, the rows correspond to COVID-19, CAP, adenocarcinoma, NTM, and TB, respectively. The columns show the original CT image and heatmaps generated by SimpleNet, PatchCore, CutPaste, CS-Flow, UTRAD, and the proposed method.

SimpleNet and PatchCore generally produce more reasonable heatmaps than CS-Flow and CutPaste in several cases, and their responses sometimes cover a larger portion of the visually abnormal lung region. Nevertheless, their localization is still not sufficiently precise. For example, in the CAP sample, both methods fail to capture the full extent of the diffuse abnormal appearance. In addition, SimpleNet shows an evident mislocalization in the adenocarcinoma example. UTRAD is a visually stronger baseline and often generates more focused activation maps compared with the other methods. However, it still shows limitations when the abnormality is spatially extensive or dispersed. In particular, for diffuse patterns such as CAP and TB, its heatmaps tend to highlight only partial regions and do not consistently cover the full visually abnormal extent.

In contrast, the proposed method shows more stable localization behavior across different pulmonary abnormality patterns. For relatively compact abnormalities, such as COVID-19, adenocarcinoma, and NTM in the displayed samples, the proposed method suppresses unrelated background responses and preserves concentrated activations around the visually suspicious regions. For more diffuse or scattered abnormalities, such as CAP and TB, the proposed method maintains broader but still controlled responses over the abnormal lung area, rather than collapsing to only a small subregion or drifting to unrelated structures. This indicates that the fusion of reconstruction residuals, transformer token responses, and quantization-distance cues improves the robustness of qualitative anomaly localization across both localized and diffuse pulmonary lesions.

Taken together, the visualizations show that the proposed framework produces spatially coherent qualitative localization maps without pixel-level supervision. The reconstruction branch captures appearance-level discrepancy, the transformer branch identifies abnormal token-level contextual responses, and the quantization-distance branch measures deviation from the learned normal feature manifold. Their fusion leads to anomaly maps that are more spatially consistent and less affected by irrelevant activation, which is consistent with the strong slice-level detection performance reported in Table 2.

### 4.6 Ablation studies

The proposed framework was analyzed from three perspectives: the reconstruction module, the transformer-based token discrimination module, and the quantization-distance module. Figure 7 provides qualitative comparisons on representative COVID-19 and cancer samples, while Table 7 summarizes the corresponding 2D slice-level quantitative results.

**Figure 7:**
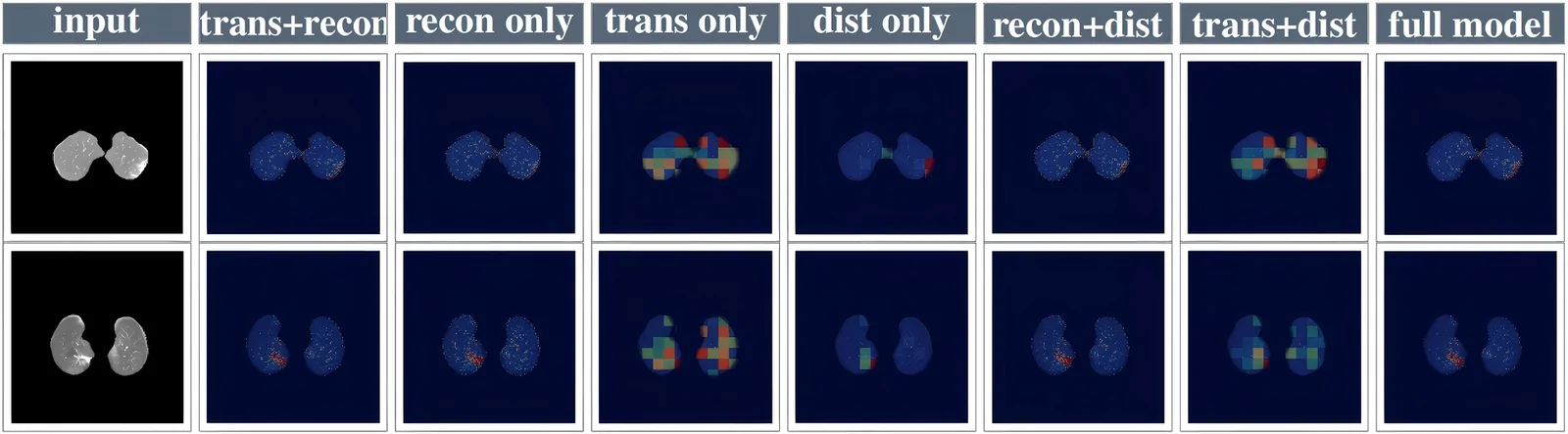
Qualitative ablation visualization of anomaly maps under different component combinations. The first row shows a representative COVID-19 sample, and the second row shows a representative cancer sample. From left to right, each row presents the input image, transformer + reconstruction, reconstruction only, transformer only, distance only, reconstruction + distance, transformer + distance, and the full model.

**Table 7:** Ablation results of different component combinations.

Setting	AUROC	Recall	F1-score	Accuracy
Reconstruction only	0.8248	0.8923	0.9315	0.8742
Transformer only	0.8484	0.8540	0.9152	0.8483
Distance only	0.8525	0.9142	0.9451	0.8981
Reconstruction + distance	0.8356	0.8984	0.9351	0.8804
Transformer + distance (λ=0.7)	0.8628	0.8754	0.9256	0.8652
Reconstruction + transformer	0.8803	0.9162	0.9473	0.9022
Reconstruction + transformer + distance (λ=0.7)	**0.9218**	**0.9611**	**0.9703**	**0.9435**

The best result in each column is shown in bold.

#### 4.6.1 Reconstruction module

The reconstruction module alone achieves an AUROC of 0.8248, a Recall of 0.8923, an F1-score of 0.9315, and an Accuracy of 0.8742, indicating that reconstruction discrepancy already captures useful abnormality information. However, it is also the weakest single-branch result in terms of AUROC. The qualitative maps in Figure 7 show that the *Reconstruction Only* setting produces relatively fine-grained responses, but the activations are scattered and noisy. This suggests that reconstruction error alone is sensitive to local appearance deviations, yet it may also respond to normal structural variations and therefore lacks sufficient specificity. When the transformer branch is introduced, the joint *Reconstruction + Transformer* setting improves AUROC from 0.8248 to 0.8803 and Accuracy from 0.8742 to 0.9022, showing that the reconstruction module benefits substantially from additional semantic discrimination.

#### 4.6.2 Transformer-based anomaly classification module

The transformer branch alone achieves an AUROC of 0.8484, which is higher than that of the reconstruction-only setting, indicating that token-level anomaly classification provides a stronger global ranking signal. In the implementation, this branch is trained with a codebook-based synthetic anomaly construction strategy: selected latent tokens are replaced by nearest-neighbor codebook features, and the transformer learns to distinguish perturbed tokens from unperturbed ones. This mechanism is not treated as an independent inference module, since its role is to provide supervision for transformer training rather than to generate a separate anomaly score at test time. Conceptually, it is not intended to make the model fit handcrafted artificial anomalies; instead, it deliberately disrupts normal latent structure so that the transformer can better learn the distinction between healthy patterns and abnormal deviations. As shown in Figure 7, the *Transformer Only* maps are semantically meaningful but spatially coarse, since they are derived from responses on a low-resolution latent token grid. This makes the transformer branch effective at highlighting suspicious regions, but insufficient by itself for precise spatial localization. When it is combined with the reconstruction branch, the resulting heatmaps become more balanced, suggesting that the transformer module contributes semantic selectivity that complements the fine-grained but noisy reconstruction cue.

#### 4.6.3 Distance module

The distance module is training-free in Stage II and is introduced only during inference as a score reweighting factor. Even by itself, *Distance Only* achieves an AUROC of 0.8525, a Recall of 0.9142, an F1-score of 0.9451, and an Accuracy of 0.8981, outperforming the other two single-branch settings on most metrics. This indicates that deviation from the learned quantized feature manifold is already a strong indicator of abnormality. Compared with reconstruction error, which is mainly measured in pixel space, the distance term is computed in latent token space by comparing encoder features with their quantized counterparts. As a result, it captures whether a local region can be well represented by the learned normal codebook, rather than only whether it can be accurately reconstructed at the image level. This makes it particularly useful when abnormal regions are partially reconstructable or when the decoder overgeneralizes subtle lesions. In Figure 7, the *Distance Only* maps are cleaner and more structured than the reconstruction-only maps, but they often emphasize only the most deviated subregions and may not fully capture lesion extent. When distance reweighting is added to reconstruction or transformer alone, the performance improves slightly, as shown by *Reconstruction + Distance* and *Transformer + Distance*. The largest gain is obtained when the distance module is integrated into the joint reconstruction-transformer model: compared with *Reconstruction + Transformer*, adding the distance term improves AUROC from 0.8803 to 0.9218, Recall from 0.9162 to 0.9611, F1-score from 0.9473 to 0.9703, and Accuracy from 0.9022 to 0.9435. A plausible reason is that the distance term provides a complementary latent normal-manifold deviation signal. It can suppress false positives in regions where the reconstruction residual is caused mainly by low-level reconstruction noise but the latent feature is still well represented by the normal codebook. At the same time, it can enhance regions where image-space reconstruction discrepancy, transformer-based token response, and codebook-representability mismatch occur together. Therefore, the distance module is most effective as an auxiliary enhancement on top of the combined reconstruction-transformer score, rather than as a standalone anomaly detector.

Overall, the ablation results suggest a clear division of roles among the three modules. The reconstruction module provides fine-grained appearance discrepancy, the transformer module provides semantic abnormality discrimination, and the distance module provides a structured measure of deviation from the normal latent manifold. Their combination yields the most compact qualitative heatmaps and the best quantitative detection performance.

#### 4.6.4 Effect of the distance weight λ

The influence of the distance weight $\lambda_{\mathrm{dist}}$ was further examined under the full fused setting, i.e., *Reconstruction* + *Transformer* + *Distance*. In this experiment, the reconstruction branch and transformer branch were jointly trained, and the final anomaly score followed Equation 33. The distance weight $\lambda_{\mathrm{dist}}$ controls the contribution of the quantization-distance cue during inference. It should be noted that $\lambda_{\mathrm{dist}}$ is only applied at the inference stage and does not modify the Stage II training objective. The evaluated values were $\lambda_{\mathrm{dist}}\in \{0.1,0.2,0.3,0.4,0.5,0.6,0.7,0.8,0.9,1.0\}$.

As shown in Table 8, the fused model is relatively stable when λ is within a moderate range, especially from 0.5 to 0.8. When λ is too small, the quantization-distance cue is underutilized and the final score mainly depends on the reconstruction-transformer response. When λ becomes too large, the distance term may over-amplify local responses and reduce robustness.

**Table 8:** Ablation results under different values of the distance weight λ in the full fused setting.

λ	AUROC	Recall	F1-score	Accuracy
0.1	0.9058	0.9482	0.9634	0.9309
0.2	0.8932	0.9471	0.9624	0.9291
0.3	0.9176	0.9576	0.9681	0.9394
0.4	0.9080	0.9500	0.9640	0.9320
0.5	0.9168	0.9584	0.9683	0.9398
0.6	0.9197	**0.9623**	**0.9704**	**0.9438**
0.7	**0.9218**	0.9611	0.9703	0.9435
0.8	0.9191	0.9522	0.9649	0.9335
0.9	0.8979	0.9473	0.9618	0.9279
1.0	0.9118	0.9557	0.9672	0.9379

The best result in each column is shown in bold.

Among all tested values, λ=0.7 achieves the highest AUROC, while λ=0.6 obtains the best Recall, F1-score, and Accuracy by a very small margin. Since AUROC is threshold-independent and is commonly used as the primary metric for anomaly detection, λ=0.7 was used as the default setting in the main experiments. The close performance between λ=0.6 and λ=0.7 also suggests that the proposed fusion strategy is not highly sensitive to minor changes in the distance weight.

## 5 Discussion

### 5.1 Summary of findings

This study presented a normal-only pulmonary CT anomaly-detection framework that combines reconstruction residuals, transformer token responses, and quantization-distance information through a shared residual vector-quantized codebook. The proposed method achieved a slice-level AUROC of 0.9218 and retained strong performance after all valid slices were aggregated at the patient level, with an AUROC of 0.9187±0.0123, balanced accuracy of 0.7521±0.0354, and F1-score of 0.9297±0.0052. Patient-level bootstrapping yielded a 95% AUROC confidence interval of 0.8786–0.9727. Compared with UTRAD, the proposed method achieved a higher observed patient-level AUROC (ΔAUROC=0.0344), while the paired bootstrap 95% confidence interval for the difference (−0.0501 to 0.1233) included zero and therefore did not establish a statistically conclusive between-method difference. Quantitative external localization on 100 consensus-selected LIDC-IDRI cases produced a P-AUROC of 0.9155. Together with the ablation study, these findings indicate that the three cues contribute complementary information at both detection and localization levels.

### 5.2 Contributions

The main contribution of this study is twofold. From the application perspective, this work studies pulmonary CT anomaly detection in a normal-only setting and evaluates the proposed framework across multiple abnormal categories, including infectious lung disease, lung cancer, and mycobacterial pulmonary disease. This demonstrates the potential of unsupervised anomaly detection as a preliminary screening tool for heterogeneous pulmonary abnormalities.

From the algorithmic perspective, this work proposes a multi-cue normality modeling framework that combines three complementary sources of anomaly evidence. The reconstruction branch captures pixel-level appearance discrepancies, the transformer branch learns token-level contextual inconsistency through codebook-based latent perturbation, and the quantization-distance branch measures deviation from the learned normal latent manifold. The experimental and ablation results show that these cues are complementary and that their fusion improves anomaly detection performance compared with using any individual cue alone.

### 5.3 Dataset-source bias and interpretation

Combining public datasets increases disease diversity but also introduces potential confounding from scanners, reconstruction kernels, slice thickness, image quality, and source-specific preprocessing. The same-source COVID-CT-MD comparisons are informative because normal, COVID-19, and CAP cases originate from one dataset; the method remained discriminative for both COVID-19 and CAP under this restriction. Disease-wise performance was also retained for NTM and TB. These findings provide no obvious pattern of failure consistent with a classifier based only on dataset identity.

Nevertheless, source bias cannot be excluded. Normal training data originated primarily from COVID-CT-MD, and matched normal controls were not available from every abnormal-data source. The LIDC-IDRI experiment provides external localization validation on an independent nodule dataset, but it is not an external patient-level classification study. A definitive assessment will require multi-institutional cohorts containing both normal and abnormal patients scanned under overlapping acquisition protocols, as well as leave-one-source-out or prospective external validation.

The current results should therefore be interpreted as retrospective methodological evidence. The framework is not clinically validated and should not be presented as a reliable diagnostic or screening system without prospective evaluation, reader studies, calibration analyses, and assessment across clinical sites.

### 5.4 Limitations and future work

First, the current framework processes 2D CT slices and does not explicitly model inter-slice continuity. A practical 2.5D extension could encode a center slice together with adjacent slices and fuse corresponding latent tokens before quantization or anomaly scoring. For example, tokens at the same in-plane location across neighboring slices could be combined using axial attention, gated feature fusion, or a lightweight sequence transformer while retaining the shared normal codebook. A full 3D extension could replace the 2D encoder with a volumetric encoder and quantize 3D latent patches, although memory-efficient codebook lookup and volumetric decoding would be required.

Second, the primary disease datasets do not provide uniform pixel-level annotations. Quantitative localization was added on 100 LIDC-IDRI cases, but this external cohort represents pulmonary nodules and does not cover the diffuse infection patterns present in COVID-19, CAP, NTM, or TB. Future work should evaluate P-AUROC, AUPRO, Dice, and intersection-over-union on broader datasets with expert lesion masks.

Third, the multi-dataset design remains vulnerable to source confounding, as discussed in Section 5.3. Future studies should include matched normal and abnormal cohorts from each institution and perform leave-one-dataset-out and prospective external validation.

Fourth, the patient-level analysis used the prespecified mean of the highest 10% of slice scores. The sensitivity analysis showed that absolute AUROC values varied across mean, maximum, and top-10% aggregation, although the proposed method retained the highest observed AUROC under all three rules. This dependence of the absolute score on the aggregation strategy remains relevant because compact and diffuse diseases may distribute abnormal slices differently across a CT volume. Future work should therefore investigate learned multiple-instance or attention-based aggregation while retaining independent normal validation for threshold calibration.

## Data Availability

Publicly available datasets were analyzed in this study. This data can be found here: https://www.cancerimagingarchive.net/collection/lung-pet-ct-dx/ https://springernature.figshare.com/articles/dataset/COVID-CT-MD_COVID-19_Computed_Tomography_Scan_Dataset_Applicable_in_Machine_Learning_and_Deep_Learning/12991592?file=26069987 https://www.kaggle.com/datasets/damianhan/nifti-dataset/data.
