# Supplementary material for: Codebook-guided multi-cue normality modeling for pulmonary CT anomaly detection
Source: Front Med (Lausanne). 2026 Sep 25;13:1927996. doi: 10.3389/fmed.2026.1927996 (PMC13649406; doi:10.3389/fmed.2026.1927996)
Supplement: Supplementary file 1 [file Datasheet1.pdf]

Supplementary Implementation and Configuration Details

Additional configuration details for the proposed pulmonary CT anomaly-detection framework and baseline models

Purpose. This supplementary document records implementation settings and model configurations that are useful for reproducibility.

1. Common experimental configuration

Item	Implementation used in this study
Split generation seed	1973
Normal patient split	80% training / 10% validation-calibration / 10% test
Abnormal patients	Reserved for testing only
Repeated-training seeds	1973, 2024, 3407
CT window	Window level -600 HU; window width 1500 HU; clipped range -1350 to 150 HU
Intensity conversion	Linear mapping of the windowed image to 8-bit grayscale [0, 255]
Lung segmentation	Pretrained lungmask R231; pixels outside the lung masks set to zero
Full-volume slice-retention filter	Foreground grayscale value > 2; retained foreground fraction >= 1%

Method-specific input resizing, channel conversion, normalization, and optimization settings are listed below. Common patient-level evaluation procedures are not repeated here.

2. Baseline model configurations

2.1 UTRAD

Item	Implementation used in this study
Backbone	ResNet-18; ImageNet-pretrained; frozen during UTRAD training.
Feature layers	Outputs from ResNet-18 layer1, layer2, and layer3 were spatially aligned/concatenated before the U-Transformer.
Trainable module	UTRAD U-Transformer reconstruction/scale-estimation network; GELU activation.
Input preprocessing	Grayscale lung-only PNG -> RGB conversion -> Resize 256x256 -> ToTensor -> ImageNet normalization (mean 0.485/0.456/0.406; std 0.229/0.224/0.225).
Training epochs	150 epochs in the lung-only repeated-training protocol.
Optimizer	Adam, betas=(0.5, 0.999).
Learning rate	1e-4.
Training batch size	8.

2.2 PatchCore

Item	Implementation used in this study
Backbone	ResNet-18 loaded with ImageNet-pretrained weights.
Feature layers	layer2 and layer3.
Input preprocessing	Grayscale lung-only PNG -> RGB -> bicubic Resize 128x128 -> ToTensor -> ImageNet normalization. Resize and final image size were both 128, so no effective center crop was applied.
Training	Non-parametric fitting only; no gradient-based epochs and no optimizer. The normal-training images were embedded once to build the PatchCore memory bank.
Training batch size	8.
Embedding dimensions	pretrain_embed_dimension=256; target_embed_dimension=256.
Patch/NN settings	Patch size 3; anomaly_scorer_num_nn=3.
Coreset	Approximate greedy coreset sampler
Nearest-neighbour search	FAISS-based nearest-neighbour scoring.
Slice anomaly score	Native PatchCore image anomaly score derived from distances to the normal memory bank.

2.3 SimpleNet

Item	Implementation used in this study
Backbone	ImageNet-pretrained ResNet-18.
Feature layers	layer2 and layer3.
Input preprocessing	Grayscale lung-only PNG -> RGB -> bicubic Resize 256x256 -> CenterCrop 224x224 -> ToTensor -> ImageNet normalization.
Embedding settings	pretrain_embed_dimension=1536; target_embed_dimension=1536; embedding_size=256; local patch size=3; patch stride=1.
Training schedule	40 meta-epochs; 4 discriminator epochs per meta-epoch; aed_meta_epochs=1.
Synthetic feature noise	Gaussian feature-space perturbation with noise_std=0.015; mix_noise=1.
Discriminator	2 layers, hidden dimension 1024, margin 0.5.
Optimizer	Discriminator: Adam with weight decay 1e-5 and cosine learning-rate schedule. One-layer pre-projection: AdamW.
Learning rate	Discriminator lr=2e-4. Configured projection lr=1e-3; repository applies lr*0.1 to the projection optimizer, giving an effective pre-projection lr=1e-4.
Training batch size	8.
Inference batch size	32 in the optimized full-volume inference implementation; this changes throughput only, not scoring.
Slice anomaly score	Native SimpleNet image score: negative discriminator output at patch level, reduced by the maximum patch anomaly score.

2.4 CS-Flow

Item	Implementation used in this study
Backbone	ImageNet-pretrained EfficientNet-B5; frozen feature extractor.
Feature extraction	Three image scales; EfficientNet-B5 feature dimension=304.
Input preprocessing	Grayscale lung-only PNG -> RGB -> Resize 256x256 -> ToTensor -> ImageNet normalization.
Flow architecture	3 scales; 4 coupling blocks; clamp=3; internal hidden width=1024; coupling kernels=[3,3,3,5].
Training schedule	5 meta-epochs x 60 sub-epochs = 300 full training epochs/passes.
Optimizer	Adam, eps=1e-4, weight_decay=1e-5; gradient clipping max norm=1.0.
Learning rate	2e-4.
Training batch size	16.
Slice anomaly score	Original multiscale CS-Flow score: 0.5 times the mean squared magnitude of concatenated flow outputs.

2.5 CutPaste

Item	Implementation used in this study
Backbone	ImageNet-pretrained ResNet-18 within ProjectionNet.
Variant	CutPaste 3-way classification: original image, standard CutPaste augmentation, and scar-like CutPaste augmentation.
Projection head	One 512-dimensional hidden projection layer followed by a 128-dimensional embedding output.
Density embedding	L2-normalized 512-dimensional backbone embedding (embed_mode=backbone).
Training preprocessing	ColorJitter (brightness=0.1, contrast=0.1, saturation=0.1, hue=0.1) -> bicubic Resize 224x224 -> CutPaste3Way -> ToTensor -> ImageNet normalization.
Evaluation preprocessing	Bicubic Resize 224x224 -> ToTensor -> ImageNet normalization; no CutPaste augmentation.
Training schedule	60 configured training epochs with 128 parameter-update steps per epoch, corresponding to 7,680 optimizer updates in total.
Optimizer	SGD, momentum=0.9, weight_decay=3e-5.
Learning rate	0.03.
Training batch size	64 normal images. Under three-way augmentation, original + two augented views are concatenated, giving an effective forward batch of 192 images.
Repeat length	3000 samples in the repeated training dataset.
Density model	GaussianDensityTorch fitted after training to L2-normalized normal-training embeddings.
Slice anomaly score	Mahalanobis distance from the fitted normal Gaussian embedding distribution; larger distance indicates greater abnormality.

3. Proposed method configuration

3.1 Encoder and latent representation

Item	Implementation used in this study
Input	1 x 128 x 128 grayscale lung-only PNG tensor.
Encoder	Modified ImageNet-pretrained ResNet-18, one-channel input.
Latent shape	512 x 16 x 16.
Number of latent tokens	256.
Token dimension	512.

3.2 Residual quantizer and codebook

Item	Implementation used in this study
Residual quantization levels	4.
Codebook size	2048 vectors.
Code vector dimension	512.
Codebook sharing	Shared across all residual levels.
Assignment	Nearest code by squared Euclidean distance.
Codebook update	Exponential moving average (EMA).
EMA decay	0.99.
EMA epsilon	1e-5.
Restart unused codes	Enabled.

3.3 Reconstruction decoder architecture

Both reconstruction decoder branches use the same three-stage topology. Stage-I decoder weights are used to initialize the Stage-II decoder branches.

Stage	Input shape	Upsampling	Convolution 1	Convolution 2	Output shape
Latent	512x16x16	-	-	-	512x16x16
Decoder block 1	512x16x16	ConvTranspose2d 512->256, k=2, s=2	3x3 Conv 256->256 + BN + ReLU	3x3 Conv 256->256 + BN + ReLU	256x32x32
Decoder block 2	256x32x32	ConvTranspose2d 256->128, k=2, s=2	3x3 Conv 128->128 + BN + ReLU	3x3 Conv 128->128 + BN + ReLU	128x64x64
Decoder block 3	128x64x64	ConvTranspose2d 128->64, k=2, s=2	3x3 Conv 64->64 + BN + ReLU	3x3 Conv 64->64 + BN + ReLU	64x128x128
Output	64x128x128	-	1x1 Conv 64->1	Sigmoid	1x128x128

3.4 Transformer/token-discrimination architecture

Item	Implementation used in this study
Token grid	16x16 = 256 tokens.
Token dimension	512.
Depth	12 residual attention blocks.
Attention heads	16.
Head dimension	32.
Attention inner dimension	512 (=16x32).
Dropout	0.1 in attention projections.
Positional embedding	Learnable 1x256x512 tensor.
Classification head	LayerNorm(512) -> Linear(512,2).
Hidden dimension	2048.

3.5 Stage-I training configuration

Item	Implementation used in this study
Training data	Normal training patients only; fixed patient split generated with split seed 1973.
Input	Single-channel 128x128.
Epochs	50.
Batch size	16.
Optimizers	Separate AdamW optimizers for encoder, residual quantizer module, and decoder.

Item	Implementation used in this study
Learning rate	1e-4 for all three optimizers.
AdamW betas	(0.5, 0.9).
Weight decay	0.0.

3.6 Stage-II training configuration

Item	Implementation used in this study
Epochs	20.
Batch size	16.
Optimizer	Adam over the trainable Stage-II decoder/transformer parameters.
Learning rate	1e-4.
Weight decay	No explicit weight decay is passed to the active Adam optimizer (PyTorch default 0).
Synthetic-token fraction	Top 10% of latent positions (approximately 25 of 256 tokens).
Perturbation mechanism	Selected latent tokens are replaced by sampled candidates from the learned shared normal codebook; 20 nearest codebook candidates are considered.
Frozen modules	Encoder and residual quantizer

3.7 Inference configuration

Item	Implementation used in this study
Primary score mode	fuse.
Distance fusion weight	dist_lambda=0.7.
Distance calibration population	Normal training slices only.
Distance standardization epsilon	1e-6.
Distance clipping	Negative standardized values clipped to 0.
Transformer-map upsampling	Nearest-neighbour to 128x128.
Distance-map upsampling in revised full-volume script	Bilinear to 128x128.
